# Supplementary material for: Allosteric regulation and crystallographic fragment screening of SARS-CoV-2 NSP15 endoribonuclease
Source: Nucleic Acids Res. 2023 Apr 28;51(10):5255–70. doi: 10.1093/nar/gkad314 (PMC10250223; doi:10.1093/nar/gkad314)
Supplement: gkad314_Supplemental_Files [file gkad314_supplemental_files.zip › Supplementary datasets.zip!/134/Refine_9/01-BUSTER/Cycle-4/LIST.html]

Refining

**BUSTER 2.X**: A suite of programs for macromolecular structure refinement by maximum-likelihood and Bayesian methods  
Copyright © 1992-2020 Global Phasing Ltd.  
All rights reserved.  
Authors: G. Bricogne, E. Blanc, M. Brandl, C. Flensburg, P. Keller, W. Paciorek, P. Roversi, O. Smart, C. Vonrhein, T. Womack  

Please reference use of the software as:

- BUSTER-TNT 2.X, Global Phasing Ltd, Sheraton House, Cambridge CB3 0AX, UK

Version: 2.10.3 for linux64-ifort made on Sep 18 2020 at 01:21:52  
Licenced to: Diamond Light Source, Scientific Software  

```
---


### Parsing of the BUSTER cardfile

TITLE : autoBUSTER refinement

   Cell information

   Symmetry information

 Using CCP4 X-ray form factors.

   Missing structure chemical composition

 Using ATOM block for chemical composition.


   Shell information

### Parsing of the TNT cardfile


---


### Reading observations file

 Number of reflexions in input:  52557

      Number of accepted reflexions:  49168

      Number of rejected reflexions:      0

   Preparing complete.mtz containing:      52505 reflexions


   Reflexions in test set:            2531

   Reflexions in working set:        46637


---


### 1. Generating initial model

 2.10 * Shannon sampling rate. NX NY NZ:   288  288  192


  Effective Shannon rates are:    2.36    2.36    2.18


   Allocated BUFFER of size:     61.172 Mb.

   Initial Geometry Information


 No missing atoms declared


   Summary of model volumes

 No missing atoms declared: no prior computed


 Computing bulk solvent model scattering


 NCSMASK PDB radius for solvent mask:     2.15 Angstrom

 running PDBSET (setting cell and spacegroup P1) ... done
 running NCSMASK [1] (creating mask, SMOOTH 1.14467) ... done
 running MAPDUMP (to check if reduction to unit cell is needed) ... done
 running MAPMASK (converting mask to map) ... done

   Generation of babslv by theta-filter

   Electron density in frg mask:  0.420 Å-1

 FOBS  put on rough absolute scale by Wilson plot :
           K        =  0.72756E+02
           B        =    -11.122

 

### Cycle 0001: MaxLik scaling and refinement


   MaxLik Scaling Cycle 0001

   Wilson plot

   Statistics in resolution bins just after scaling
```

|  |  |
| --- | --- |
|  | - Rxpct factors plot- Rxpct factors table- <Log-Lik Gain>- Ln Sigma A- Correlation Coefficients |

```
   <Log-Likelihood> of null-hypothesis

Structure Factor Amplitudes


   Average Figure of Merit

 Switching off refinement of overall temperature factor


 Added B =    -0.4402 Å2 to all atoms in fragment


 Added B =    -0.4402 Å2 to B_SOLV

```
gelly refinement module
 Authors (2004-2009) O. S. Smart, C. Flensburg, W. Paciorek, C. Vonrhein, 
                     M. Brandl, T. O. Womack and G. Bricogne
         (2010-2011) O. S. Smart, C. Flensburg, W. Paciorek, C. Vonrhein, 
                     T. O. Womack and G. Bricogne
         (2012-2014) O. S. Smart, C. Flensburg, W. Paciorek, C. Vonrhein, 
                     and G. Bricogne
         (2015-2020) G. Bricogne, C. Flensburg, W. Paciorek, C. Vonrhein
 Copyright © 2004-2020 Global Phasing Ltd. 
 All rights reserved.
 gelly module compilation date Sep 18 2020 01:18:35
 
 gelly uses routines from TNT geometry. References: 
     Tronrud, D. E., Ten Eyck, L. F., Matthews, B. W. (1987)
     "An Efficient General-Purpose Least-Squares Refinement Program 
     for Macromolecular Structures". Acta Crystallogr A, 43:489-501.
     Tronrud, D. E. (1997). "The TNT Refinement Package", 
     in Macromolecular Crystallography, Part B, Eds Charlie Carter 
     and Robert Sweet, Methods in Enzymology, 277:306-319.
 wildcard matching function by Julian Robichaux http://www.nsftools.com

 No -random_seed flag found. Random number stream initialised using:
2147483562
2147483398

 Control flags set to:  -autoncs -hydrogens_input_occ2zero \
                     -hydrogens_output_occ2heavy -screen \
                     100 -screen_sigma \
                     3.0 -keeppdb \
                     1000000 -taomonitor_verbose \
                     2 
 

 ############### Starting setup via TNT routines

 Setting up tnt routines including license check:

                   TNT Stereochemistry Program
 Version: 5.22.3.0
 Licence for Diamond Light Source, Scientific Software is valid
 
 Using TNT geometry routines to load the tnt control file: gelly.tnt
 Using TNT routines to load initial coordinates from file fragment.cor
 Expecting 6202 atoms

 Found     2191 "NOTE BUSTER_RESIDUE_ATOM_DATA" cards
 Have copied information for    6202 atoms from TNT to gelly's store 
 
 The TNT WEIGHT cards used by this run:
 -----WEIGHT       BOND     2.0
 -----WEIGHT       ANGLE    2.0
 -----WEIGHT       IMPROPER 0.0
 -----WEIGHT       TORSION  2.0
 -----WEIGHT       PSEUDO   0.0
 -----WEIGHT       TRIGONAL 2.0
 -----WEIGHT       PLANE    5.0
 -----WEIGHT       CONTACT  5.0
 -----WEIGHT       BCORREL  10.0
 -----WEIGHT       CHIRAL   5.0
 -----WEIGHT       NCS      50.0
 -----WEIGHT       IDEAL    4.0
 -----WEIGHT       RFACTOR  9.34
 
 Dictionary versions picked up from NOTE BUSTER_RESTRAINT_DICT cards as: 
 ------ protgeo_eh99.dat (V1.6.4.17) 20191001  STANDARD AMINO ACID DICTIONARY. BONDS AND ANGLES FROM ENGH AND HUBER EH99. OTHER VALUES BASED ON PREVIOUS TNT OR TAKEN FROM CCP4. INCLUDES HYDROGEN ATOMS.
 ------ exoticaa.dat (V1.3.4.15) 20200327  COLLECTION OF NON-STANDARD AMINO ACIDS, MAINLY EH91 WITHOUT IDEAL DISTANCE INFO
 ------ nuclgeo.dat (V1.13.4.8) 20190909
 ------ bcorrel.dat (V1.15.10.2) 20190616
 ------ contact.dat (V1.15.12.10) 20191121
 ------ idealdist_contact.dat (V1.3.4.12) 20191001  IDEAL-DISTANCE CONTACT TERM DATA AS USED IN PROLSQ. VALUES USED HERE ARE BASED ON DATA HARVESTING OF PDB AS OF 20190109.
 ------ restraints for LIG (Unknown) from cif dictionary Z1713595338.cif
 ------ restraints for CIT (CITRIC ACID) from cif dictionary CIT.cif; generated by GRADE_PDB_LIGAND 1.2.12 (October 4 2016) using MOGUL 1.7.2(RC1), CSD as538be, with quantum mechanics RM1 ((DatabaseID PDB-CIT))
 ------ assume.dat (V1.9.4.2) 20180705
 
 Have picked up spacegroup name for pdb output as 'P 63      '
 Setting up Chain/Residue arrays
 
 Have command line specification -autoncs*
     Have found       1 NCS relations
     Pairwise matching residue number and type between chains: 
                    A        B        S        W    
          A         .       99%       -        -   
          B                  .        -        -   
          S                           .        -   
          W                                    .   
       Table key--                                                       
          all   : two chains have 100% match and same number of residues 
         multi  : ditto and they contribute to a multi chain ncs cluster 
          ***%  : two chains have >80% matching residue number/type      
           -    : chains appear to be unrelated                          
      chain A     have identified ncs to     1 other chains. So will weight each autoncs by (       6.666/#)=      6.6660
      chain B     have identified ncs to     1 other chains. So will weight each autoncs by (       6.666/#)=      6.6660
      chain S     have identified ncs to     0 other chains. 
      chain W     have identified ncs to     0 other chains. 
     For .Gelly cards produced see below - after pruning
 
 Setting up BUSTER_SET groups
 Have found    91 BUSTER_SET cards
 Now silently applying automatic/dictionary set definitions. For a verbose listing use '-verbose_set' flag.
 Applying user defined set definitions:
 =====NOTE BUSTER_SET Ligand = { B|501 } --> set 'Ligand' now has    11 atoms 
 =====NOTE BUSTER_SET NotLigand = \ Ligand --> set 'NotLigand' now has  6191 atoms 
 =====NOTE BUSTER_SET ncsautoXcld = Water --> set 'ncsautoXcld' now has   662 atoms 
 =====NOTE BUSTER_SET ncsautoABset = Chain_A \ ncsautoXcld --> set 'ncsautoABset' now has  2771 atoms 
 Have defined    51 sets: 
      set:                 'All' has   6202 atoms in   1362 residues - automatically defined
      set:               'Empty' has      0 atoms in      0 residues - automatically defined
      set:            'ExoticAA' has      0 atoms in      0 residues - defined by dictionary file
      set:           'ExoticNUC' has      0 atoms in      0 residues - defined by dictionary file
      set:              'NotHET' has   5516 atoms in    698 residues - defined by dictionary file
      set:             'Chain_A' has   2771 atoms in    350 residues - automatically defined
      set:             'Chain_B' has   2769 atoms in    350 residues - automatically defined
      set:             'Chain_S' has    373 atoms in    373 residues - automatically defined
      set:             'Chain_W' has    289 atoms in    289 residues - automatically defined
      set:               'Water' has    662 atoms in    662 residues - defined by dictionary file
      set:          'StdProtein' has   5516 atoms in    698 residues - defined by dictionary file
      set:             'Protein' has   5516 atoms in    698 residues - defined by dictionary file
      set:                'Back' has   2794 atoms in    698 residues - defined by dictionary file
      set:                'Side' has   2722 atoms in    652 residues - defined by dictionary file
      set:        'RnaCanonical' has      0 atoms in      0 residues - defined by dictionary file
      set:        'DnaCanonical' has      0 atoms in      0 residues - defined by dictionary file
      set:     'RnaDnaCanonical' has      0 atoms in      0 residues - defined by dictionary file
      set:     'DnaRnaCanonical' has      0 atoms in      0 residues - defined by dictionary file
      set:         'RnaExtended' has      0 atoms in      0 residues - defined by dictionary file
      set:         'DnaExtended' has      0 atoms in      0 residues - defined by dictionary file
      set:      'RnaDnaExtended' has      0 atoms in      0 residues - defined by dictionary file
      set:      'DnaRnaExtended' has      0 atoms in      0 residues - defined by dictionary file
      set:                 'Dna' has      0 atoms in      0 residues - defined by dictionary file
      set:                 'Rna' has      0 atoms in      0 residues - defined by dictionary file
      set:              'RnaDna' has      0 atoms in      0 residues - defined by dictionary file
      set:              'DnaRna' has      0 atoms in      0 residues - defined by dictionary file
      set:             'Nucleic' has      0 atoms in      0 residues - defined by dictionary file
      set:             'Polymer' has   5516 atoms in    698 residues - defined by dictionary file
      set: 'BufferWeichenberger' has     13 atoms in      1 residues - defined by dictionary file
      set:    'BufferAdditional' has      0 atoms in      0 residues - defined by dictionary file
      set:              'Buffer' has     13 atoms in      1 residues - defined by dictionary file
      set:         'MetalAlkali' has      0 atoms in      0 residues - defined by dictionary file
      set:  'MetalAlkalineEarth' has      0 atoms in      0 residues - defined by dictionary file
      set:     'MetalTransition' has      0 atoms in      0 residues - defined by dictionary file
      set: 'MetalPostTransition' has      0 atoms in      0 residues - defined by dictionary file
      set:    'MetalLanthanides' has      0 atoms in      0 residues - defined by dictionary file
      set:               'Metal' has      0 atoms in      0 residues - defined by dictionary file
      set:         'SugarAlphaD' has      0 atoms in      0 residues - defined by dictionary file
      set:         'SugarAlphaL' has      0 atoms in      0 residues - defined by dictionary file
      set:               'Sugar' has      0 atoms in      0 residues - defined by dictionary file
      set:               'Other' has     24 atoms in      2 residues - defined by dictionary file
      set:            'Hydrogen' has      0 atoms in      0 residues - defined by dictionary file
      set:         'nonHydrogen' has   6202 atoms in   1362 residues - defined by dictionary file
      set:             'OccZero' has      0 atoms in      0 residues - defined by dictionary file
      set:          'OccNonZero' has   6202 atoms in   1362 residues - defined by dictionary file
      set:             'OccFull' has   6159 atoms in   1359 residues - defined by dictionary file
      set:          'OccNonFull' has     43 atoms in      3 residues - defined by dictionary file
      set:              'Ligand' has     11 atoms in      1 residues - user specified
      set:           'NotLigand' has   6191 atoms in   1361 residues - user specified
      set:         'ncsautoXcld' has    662 atoms in    662 residues - user specified
      set:        'ncsautoABset' has   2771 atoms in    350 residues - user specified
 No command line arguments '-occ_allhet' or '-occ_restyp' so will not setup automated occcupancy refinement
 
 Interpreting CONSTANT and FREE cards: 
 =====NOTE BUSTER_CONSTANT OCC NotLigand
 Have found and interpreted      1 CONSTANT cards in the TNT input. 
 Before any COMBINE - Number of atoms with fixed XYZ =     0
 Before any COMBINE - Number of atoms with fixed B   =     0
 Before any COMBINE - Number of atoms with fixed OCC =  6191
 Before any COMBINE - Number of atoms with fixed BIJ =   662
                            cf total number of atoms =  6202
 
 Have a total of     2 COMBINE and/or NOTE BUSTER_COMBINE cards in TNT input
 
 There are no NOTE BUSTER_COMBINE_RES cards to be found in the TNT card input 
 Interpreting COMBINE cards: 
 =====NOTE BUSTER_COMBINE OCC Ligand
 =====NOTE BUSTER_COMBINE B Ligand
 Do not have any COMBINE groups of type XYZ
 Have    1 COMBINE groups of type B
 B   combine group number     1 has    11 atoms in it:
        Residue    B|501   LIG  C01   S02   O03   O04   N05   C06   C07   C08   C09   C10   N11    
 Have    1 COMBINE groups of type OCC
 OCC combine group number     1 has    11 atoms in it:
        Residue    B|501   LIG  C01   S02   O03   O04   N05   C06   C07   C08   C09   C10   N11    
 Number of atoms with fixed/combined XYZ =     0
 Number of atoms with fixed/combined B   =    11
 Number of atoms with fixed/combined OCC =  6202
 Number of atoms with fixed/combined BIJ =   662
                cf total number of atoms =  6202
 

 ############### Working out full connectivity list


 ############### Working out equivalent atoms


 Residue with most atoms given below:
        A|58:N(TRP)         
        A|58:CA(TRP)        
        A|58:CB(TRP)        
        A|58:CG(TRP)        
        A|58:CD1(TRP)       
        A|58:NE1(TRP)       
        A|58:CE2(TRP)       
        A|58:CD2(TRP)       
        A|58:CE3(TRP)       
        A|58:CZ3(TRP)       
        A|58:CH2(TRP)       
        A|58:CZ2(TRP)       
        A|58:C(TRP)         
        A|58:O(TRP)         


 Have no '-equiv_check_plus' argument - so will take atom type into
 account when checking for equivalence of otherwise identical atoms.
 

 ############### Looking at dihedrals


 ############### Looking at chirals

 NOTE BUSTER_CHIRAL_ANGLECUT card found - read value=    -31.00 degs from it
 NOTE BUSTER_CHIRAL_SIGMA card found - read value=      1.70 degs from it
 RANGE allowed for B variables in refinement:   from      3.00000 to    300.00000

 ############### Preparing for PDB output


 ############### Processing extra restraints explicitly defined

 No 'NOTE BUSTER_TARGET' cards or -target?? args found (used to read coordinates files for soft ncs to external)
 No 'NOTE BUSTER_OCCSUM'     cards found - so not using sum of occupancy restraints
 No 'NOTE BUSTER_DISTANCE'   cards found - so not setting up any additional atom-atom distance restraints
 No 'NOTE BUSTER_UTILANGLE'  cards found - so not setting up any additional restraints on any angle
 No 'NOTE BUSTER_UTILTOR'    cards found - so not setting up any additional sinusoidal restraints on torsions
 
 --- Have some similarity definitions 
     Similarity restraints on RMSD use superposition routines from: 
     Coutsias, E.A., Seok, C., Dill, K.A.(2004)
     "Using quaternions to calculate RMSD", J. Comput. Chem., 25:1849-1857 

 Have found    1 'NOTE BUSTER_SIM_DEFINE' cards 
     Maximum number of chains involved in any SIM group (excluding the template)=     1


 ########## Interpreting card: NOTE BUSTER_SIM_DEFINE ncsautoAB ncsautoABset B
     Number of atoms in template set=  2771
     Number of related chains (other than template) in SIM set=    1
     List of chains to be restrained (excluding template)=   B   
     Pairwise RMSD, XYZ in Angstroms, for SIM group: ncsautoAB
                 A         B   
     A         -----     ----- 
     B         0.576     ----- 
     Overall RMSD,XYZ for the SIM group is     0.576 Angstroms
     Pairwise rms deviation of [individual B's minus their set/chain average] in Angs**2
                 A         B         Bavg
     A         -----     -----     91.77
     B         22.64     -----     76.13
     Overall RMSD,B   for the SIM group is    22.640 Angs**2
     The number of common atoms for the above fits:
                 A         B   
     A         -----     ----- 
     B          2758     ----- 
     (compared to the template set that has    2771 atoms in it)
     List of atoms that are absent in one or more chains for this group: 
                                    A    B   
                    C1      401     +    -
                    O1      401     +    -
                    O2      401     +    -
                    C2      401     +    -
                    C3      401     +    -
                    O7      401     +    -
                    C4      401     +    -
                    C5      401     +    -
                    O3      401     +    -
                    O4      401     +    -
                    C6      401     +    -
                    O5      401     +    -
                    O6      401     +    -
     (key  +  atom present for this chain 
           -  atom absent  for this chain)
 transforms for each of the superpositions orthogonal matrix r11 r12 r13 r21 r22 r23 r31 r32 r33 tx ty tz (PDBSET syntax): 
TRANS_AB -0.877374 -0.479514 -0.016754 -0.479592  0.877491  0.000771  0.014332  0.008711 -0.999859    33.887    32.920    15.166
 reverse transforms 
TRANS_BA -0.877374 -0.479592  0.014332 -0.479514  0.877491  0.008711 -0.016754  0.000771 -0.999859    45.302   -12.769    15.707
 
 Setting up LSSR interatomic distance restraints
 Interpreting card: NOTE BUSTER_SIM_RESTRAIN_LSSR ncsautoAB 6.666
        setting up interatomic distance pair restraints with:
        Dmin=         0.00
        Dmax=         5.50
        sigma=        0.20
        Vmax=         3.00
        weight=       6.67
        number of distance pairs defined for this set is      34780
 Have not supplied either '-sim_swap_equiv' or '-sim_swap_equiv_plus' argument 
   so will not try swaping equivalent atoms to improve NCS and/or target agreement.
 
 NCS by LSSR outlier analysis
 -autoncs option automatic NCS prune routine -------- 
 Residues pruned out by gradient condition:
          A|135     A|140     A|148     A|149     A|151     A|181     A|182
          A|184     A|185     A|186     A|189     A|216     A|217     A|218
          A|219     A|220     A|234     A|235     A|242     A|243     A|244
          A|245     A|247     A|249     A|255     A|256     A|280     A|282
          A|285     A|286     A|287     A|288     A|289     A|317     A|318
          A|320     A|321     A|322     A|325     A|326     A|327     A|328
          A|329     A|330     A|340     A|341     A|342     A|343     A|344
          B|135     B|140     B|148     B|149     B|151     B|181     B|182
          B|184     B|185     B|186     B|189     B|216     B|217     B|218
          B|219     B|220     B|234     B|235     B|242     B|243     B|244
          B|245     B|247     B|249     B|255     B|256     B|280     B|282
          B|285     B|286     B|287     B|288     B|289     B|317     B|318
          B|320     B|321     B|322     B|325     B|326     B|327     B|328
          B|329     B|330     B|340     B|341     B|342     B|343     B|344
       
 Residues pruned out by function/max condition:
          A|149!    A|187     A|188     A|220!    A|319     A|326!    A|346 
         A|ENDA     B|149!    B|187     B|188     B|220!    B|319     B|326!
          B|346    B|ENDA 
  
 Total number of residues for which LSSR restraints have been pruned out is      108 out of      698 participating
 Number of LSSR restraints pruned out is      7422 this is   21.3% of the total           34780
  
 -autoncs .Gelly cards suitable for adaptation for manual definition/pruning
           (note auto pruning is done in a slightly different way so exact results may differ slightly)
 -autoncs option .Gelly cards also written to file  auto_gelly_cards.txt
NOTE BUSTER_SET ncsautoXcld = Water    # card added - produced by -autoncs
NOTE BUSTER_SET ncsautoXcld = ncsautoXcld + { A|135 A|140 A|148 A|149 A|151 }    # card added - produced by -autoncs
NOTE BUSTER_SET ncsautoXcld = ncsautoXcld + { A|181 A|182 A|184 A|185 A|186 }    # card added - produced by -autoncs
NOTE BUSTER_SET ncsautoXcld = ncsautoXcld + { A|187 A|188 A|189 A|216 A|217 }    # card added - produced by -autoncs
NOTE BUSTER_SET ncsautoXcld = ncsautoXcld + { A|218 A|219 A|220 A|234 A|235 }    # card added - produced by -autoncs
NOTE BUSTER_SET ncsautoXcld = ncsautoXcld + { A|242 A|243 A|244 A|245 A|247 }    # card added - produced by -autoncs
NOTE BUSTER_SET ncsautoXcld = ncsautoXcld + { A|249 A|255 A|256 A|280 A|282 }    # card added - produced by -autoncs
NOTE BUSTER_SET ncsautoXcld = ncsautoXcld + { A|285 A|286 A|287 A|288 A|289 }    # card added - produced by -autoncs
NOTE BUSTER_SET ncsautoXcld = ncsautoXcld + { A|317 A|318 A|319 A|320 A|321 }    # card added - produced by -autoncs
NOTE BUSTER_SET ncsautoXcld = ncsautoXcld + { A|322 A|325 A|326 A|327 A|328 }    # card added - produced by -autoncs
NOTE BUSTER_SET ncsautoXcld = ncsautoXcld + { A|329 A|330 A|340 A|341 A|342 }    # card added - produced by -autoncs
NOTE BUSTER_SET ncsautoXcld = ncsautoXcld + { A|343 A|344 A|346 A|ENDA }    # card added - produced by -autoncs
NOTE BUSTER_SET ncsautoABset = Chain_A \ ncsautoXcld    # card added - produced by -autoncs
NOTE BUSTER_SIM_DEFINE ncsautoAB ncsautoABset B    # card added - produced by -autoncs
NOTE BUSTER_SIM_RESTRAIN_LSSR ncsautoAB 6.666    # card added - produced by -autoncs

 LSSR function contribution before pruning     49196.5
                             after pruning     10588.3
 Graph of which residues pruned from LSSR: ncs_pruned.mtv
 Graph of LSSR function outliers ncs_function_outliers.mtv
 Graph of LSSR gradient outliers ncs_gradient_outliers.mtv
 LSSR - analysis of distance difference distribution for NCS
         DeltaD less than 0.5 Angs:  proportion of population= 98.49% average DeltaD of subset     0.013 Angs
 Histogram of LSSR deltaD distribution ncs_histogram.mtv
 
 Interpreting card: NOTE BUSTER_TLS_SET tls1 { A|* }
 Interpreting card: NOTE BUSTER_TLS_SET tls2 { B|* }
 Interpreting card: NOTE BUSTER_TLS_SET tls1 { A|* }
 Interpreting card: NOTE BUSTER_TLS_T   tls1     -3.26149E-01 -2.32596E-01  4.45630E-01 -3.91806E-02  3.95837E-02  4.09452E-02
 Interpreting card: NOTE BUSTER_TLS_L   tls1      1.68403E+00  1.68502E+00  1.03262E+00  3.16405E-01 -6.37610E-01  4.28922E-01
 Interpreting card: NOTE BUSTER_TLS_S   tls1      2.83610E-01 -2.29329E-01  2.89686E-01 -1.27619E-01 -5.00525E-02 -1.81171E-01  2.02995E-02 -1.16203E-01
 Interpreting card: NOTE BUSTER_TLS_O   tls1     -2.33284E+01 -8.36882E+01  1.12128E+01
 Interpreting card: NOTE BUSTER_TLS_SET tls2 { B|* }
 Interpreting card: NOTE BUSTER_TLS_T   tls2     -3.93889E-01 -3.24897E-01  5.24628E-01  1.56106E-02  2.30904E-02 -3.24036E-02
 Interpreting card: NOTE BUSTER_TLS_L   tls2      1.80298E+00  1.67200E+00  9.38839E-01  7.03942E-01 -9.42582E-01 -8.62192E-01
 Interpreting card: NOTE BUSTER_TLS_S   tls2     -3.02785E-02  1.54983E-01 -1.28845E-01  1.24089E-01  2.68450E-01  6.71414E-02 -1.03280E-01  6.94089E-02
 Interpreting card: NOTE BUSTER_TLS_O   tls2      2.20360E+01 -7.19286E+01  3.53859E+00
 Interpreting card: NOTE BUSTER_TLS_FIX tls1 ALL
 Interpreting card: NOTE BUSTER_TLS_FIX tls2 ALL


 TLS information. Total number of groups:     2


 Group   Natoms    FixRB   FixTLS   Group Tag    Group Spec
     1     2771      Y        Y     tls1    { A|* }
     2     2769      Y        Y     tls2    { B|* }
 
NOTE BUSTER_TLS_SET tls1 { A|* }
NOTE BUSTER_TLS_T   tls1     -3.26149E-01 -2.32596E-01  4.45630E-01 -3.91806E-02  3.95837E-02  4.09452E-02
NOTE BUSTER_TLS_L   tls1      1.68403E+00  1.68502E+00  1.03262E+00  3.16405E-01 -6.37610E-01  4.28922E-01
NOTE BUSTER_TLS_S   tls1      2.83610E-01 -2.29329E-01  2.89686E-01 -1.27619E-01 -5.00525E-02 -1.81171E-01  2.02995E-02 -1.16203E-01
NOTE BUSTER_TLS_O   tls1     -2.33284E+01 -8.36882E+01  1.12128E+01
NOTE BUSTER_TLS_FIX tls1 ALL
 
NOTE BUSTER_TLS_SET tls2 { B|* }
NOTE BUSTER_TLS_T   tls2     -3.93889E-01 -3.24897E-01  5.24628E-01  1.56106E-02  2.30904E-02 -3.24036E-02
NOTE BUSTER_TLS_L   tls2      1.80298E+00  1.67200E+00  9.38839E-01  7.03942E-01 -9.42582E-01 -8.62192E-01
NOTE BUSTER_TLS_S   tls2     -3.02785E-02  1.54983E-01 -1.28845E-01  1.24089E-01  2.68450E-01  6.71414E-02 -1.03280E-01  6.94089E-02
NOTE BUSTER_TLS_O   tls2      2.20360E+01 -7.19286E+01  3.53859E+00
NOTE BUSTER_TLS_FIX tls2 ALL


 TLS: mmCIF group specification begin


#
loop_
_pdbx_refine_tls_group.id
_pdbx_refine_tls_group.refine_tls_id
_pdbx_refine_tls_group.beg_PDB_ins_code
_pdbx_refine_tls_group.beg_auth_asym_id
_pdbx_refine_tls_group.beg_auth_seq_id
_pdbx_refine_tls_group.end_PDB_ins_code
_pdbx_refine_tls_group.end_auth_asym_id
_pdbx_refine_tls_group.end_auth_seq_id
_pdbx_refine_tls_group.selection
_pdbx_refine_tls_group.selection_details
   1   1 . A   -1 . A  346 . '{ A|* }'
   2   1 . A  401 . A  401 . '{ A|* }'
   3   2 . B   -1 . B  346 . '{ B|* }'
   4   2 . B  501 . B  501 . '{ B|* }'
#


 TLS: mmCIF group specification end


 Interpreting card: NOTE BUSTER_ANISO_SET Empty
 Interpreting card: NOTE BUSTER_ANISO_WEIGHT SCA  16.0
 Interpreting card: NOTE BUSTER_ANISO_WEIGHT SPH   0.6    0.6
 Interpreting card: NOTE BUSTER_ANISO_WEIGHT SIM   7.8    4.8    2.6    0
 Interpreting card: NOTE BUSTER_ANISO_WEIGHT RIG  21.5   21.5    3.7    0


 After TLS and ANISO: Number of atoms with fixed/combined xyz =     0
 After TLS and ANISO: Number of atoms with fixed/combined B   =    11
 After TLS and ANISO: Number of atoms with fixed/combined OCC =  6202
 After TLS and ANISO: Number of atoms with fixed/combined BIJ =  6202
 After TLS and ANISO: Number of atoms in a TLS group          =  5540
                                     cf total number of atoms =  6202
 

 ############### Setup state vector

 No free_vect_ulim_xyz argument found: using default of  100.000
 No free_vect_llim_xyz argument found: using default of -100.000
 Number of parameters added for B combine groups                =       1
 Number of parameters added for OCC combine groups              =       1
 Number of parameters added for TLS description                 =       0
 Number of parameters to describe molecular conformation        =   24799

 ############### Setup free vector

 Number of free parameters to be adjusted in refinement   =   24799

 ############### Setting up own geometry


 No command line argument '-tnt_fn' so will use gelly to do geometry function calculations

 ============= Call TNT functions


 ============= Get TNT symmetry information

 
      Getting symmetry operators from TNT. 
          gelly will classify symmetry using pdb-like convention: 
               SYMOP   SYMMETRY
              NNNMMM   OPERATOR
                1555   X,Y,Z
                2555   -Y,X-Y,Z
                3555   -X+Y,-X,Z
                4555   -X,-Y,1/2+Z
                5555   Y,-X+Y,1/2+Z
                6555   X-Y,X,1/2+Z
          where NNN -> operator number and MMM -> translation vector
 

 ============= Own Gelly geometry/restraints setup


 ----------- Identify bond-types


    Setting up GEOMBONDREC_TYPE_HASH* to be able to map a given bond type (e.g.
    "single") to an internal integer (GBOND_TYPE=1) etc.

    Using default  GEOMBONDREC_TYPE_HASH_STR='SING* 1 DOUB* 2 TRIP* 3 AROM* 4 DELO* 4 METAL* 5':
    to override default use a NOTE BUSTER_GEOMBONDREC_TYPE_HASH_STR gelly card.

 ----------- Classify torsion into peptide omega and other


 ----------- Setup of non-bonded terms

 Ideal-distance contact term setup:
    Restraint function constants (normally these values are from NOTE BUSTER_IDEAL_CONTACT_* cards)
    Sigma for general contacts      0.200 Angs
    Sigma for 1-4 contacts          0.100 Angs (contacts across a torsion)
    Sigma for 1-5 contacts          0.150 Angs
    Sigma for 1-N contacts          0.200 Angs
    Sigma for HBOND pairs           0.100 Angs (contacts between HB_types A and D or B's)
    Contact distance increment for each atom in HBOND          -0.150 Angs 
    Contact distance increment for H-bond acceptor              0.100 Angs 
    Contact distance increment for 1-4 with an O atom          -0.050 Angs 
    Contact distance increment for 1-4 with an N atom          -0.180 Angs 
    Contact distance increment for 1-4 with an C atom          -0.370 Angs 
    Contact distance increment for 1-4 with an S atom          -0.150 Angs 
    Contact distance increment for 1-4 with an P atom          -0.150 Angs 
    Contact distance increment for 1-4 with any other atom     -0.150 Angs 
    All atoms matched for CCP4 atom type.
    Number of CCP4 atom types used is         25 (H-bound and not H-bound counted separately)

 Setting up short/ideal-distance contacts
   Using gelly special position handling. (For TNT instead rerun with argument '-special_tnt')

   No command line argument '-special_all' specified. We will auto-generate
   EXCLUDE cards only for atom-atom contacts across symmetry (i.e. on special
   position) when this involves a contact within a residue to itself.

   Have set up non-bonded exclusions - 1-3 contacts
   Atom A|27:CA         makes the most 1-3 contacts:  6

   Have set up non-bonded exclusions - 1-4 contacts
   Atom A|27:N          makes the most 1-4 contacts:  7

   Have set up non-bonded exclusions - 1-5 contacts
   Atom A|28:C.A        makes the most 1-5 contacts: 10

 ############################################################################################################
 ###################################################### setting up contact terms
 ############################################################################################################

 WARNING . atom W|174:O(HOH)  has occupancy 1.0 but is at a special position?
 WARNING .EXCLUDE W|174 W|174
 WARNING . Generating this EXCLUDE card because an atom W|174:O(HOH) has a short contact to a 
 WARNING . symmetry copy of itself by operator:  2445 dist    0.347 Angs. occ 1.000
 WARNING . The card should ensure no bad contacts for the complete residue. If atom is at special position.... 


 ############################################################################################################
 ###################################################### setting up contact terms
 ############################################################################################################


 ============= Call Gelly versions of TNT functions

 WARNING: 1-4 contact without 1-(2,3)-4 data for A|401:O2(CIT)        and A|401:C3(CIT)        X-X
 WARNING: 1-4 contact without 1-(2,3)-4 data for A|401:C2(CIT)        and A|401:O6(CIT)        X-X
 WARNING: 1-4 contact without 1-(2,3)-4 data for A|401:O7(CIT)        and A|401:O5(CIT)        X-X
 WARNING: 1-4 contact without 1-(2,3)-4 data for A|401:C4(CIT)        and A|401:O6(CIT)        X-X

       TERM      weight      number      TNT_fn    Gelly_fn        diff
       BOND       2.000        5655    1755.285    1755.285       0.000
       ANGL       2.000        7677    5533.871    5533.871       0.000
       IMPR       0.000           0       0.000       0.000       0.000
       TORS       2.000        1965    3593.108    3593.108      -0.000
       TRIG       2.000           0       0.000       0.000       0.000
       PLAN       5.000         962    2481.844    2481.844       0.000
       CONT       5.000           0     352.539       0.000     352.539
       BCOR      10.000        5632    6726.710    7837.619   -1110.909


 ============= Call Gelly geometry functions

   Switching torsion functional form to sinusoidal rather than split harmonic functional.
      if you want this not to happen then rerun with '-torsharm'
      Weight for sinusoidal torsion=     2.000
   BCOR function/gradient will be split between H and non-H atoms (as for any atom pair). Use '-bcorrel_all_in_hydrogens' to change this.

 ----------- Fix B-correlation restraints

 
 Checking for bonds that do not have a bcorrel restraint defined
 We will wipe all existing BCORrels if OCC=0.0 atom(s) is/are involved. This can be' switched off via the '-bcorrel_nowipe_zeroocc' flag.
 We will wipe all existing BCORrels if only non-hydrogen atom(s) with fixed B(iso) are involved. This can be switched off via the '-bcorrel_nowipe_fixb_nonhydrogens' flag.
 We will keep all existing BCORrels if hydrogen atom(s) with fixed B(iso) is/are involved. This can be switched off via the '-bcorrel_wipe_fixb_hydrogens' flag.
 No -bcorrel_missing_sigma found. So will set sigma to     2.5 Ang**2 for any bond with no existing BCORrel
 No -bcorrel_missing_sigma_hydrogens found. So will set sigma to     1.0 Ang**2 for any bond with no existing BCORrel involving hydrogens

 No active BCORels found involving OCC=0.0 atom(s)


 No active BCORels found involving fixed-B (only) non-hydrogen atom(s)

    add a default BCOR for bond A|401:C1(CIT) A|401:C2(CIT)
    add a default BCOR for bond A|401:C1(CIT) A|401:O1(CIT)
    add a default BCOR for bond A|401:C1(CIT) A|401:O2(CIT)
    add a default BCOR for bond A|401:C2(CIT) A|401:C3(CIT)
    add a default BCOR for bond A|401:C3(CIT) A|401:C4(CIT)
    add a default BCOR for bond A|401:C3(CIT) A|401:C6(CIT)
    add a default BCOR for bond A|401:C3(CIT) A|401:O7(CIT)
    add a default BCOR for bond A|401:C4(CIT) A|401:C5(CIT)
    add a default BCOR for bond A|401:C5(CIT) A|401:O3(CIT)
    add a default BCOR for bond A|401:C5(CIT) A|401:O4(CIT)
    add a default BCOR for bond A|401:C6(CIT) A|401:O5(CIT)
    add a default BCOR for bond A|401:C6(CIT) A|401:O6(CIT)

 Have added a total of        12 BCORrels for bonds which do not already have a BCORrel


 Have skipped a total of        11 bonds to setup BCORrels where both atoms have constant/fixed B(iso)


 ----------- Deactivate hydrogen-free planes if hydrogen atoms present

 A total of       0 planes have been deactivated because they are duplicated hydrogen-free and with-hydrogen forms.

 ----------- Handle peptide omega angle(s)

      Torsion angles split into peptide omega and other
         Number of peptide omega angles is       698
         Number of other torsion is             1267
      Peptide omega angles are monitored rather than directly restrained (PLANE used instead).

 ----------- Fix cis-PRO

 Now apply the fix to get correct restraints for cis PRO residues
   Have got      11 bonds records and      26 angles
   from GEOMETRY cards for the special PROC residue
   Weight for ideal-distance contact term=     4.000

 ----------- Prepare SSBOND/LINK for output PDB file


 ----------- Prepare for QM


 ----------- Prepare symmetry in utility distance restraints


 ############### Setting up Rhofit contacts (if needed)

 No ligand-(fixed protein) contact term will be used

 ############### Working out initial function and gradient


   MaxLik Scaling Cycle 0002
 
 Initial functional value = 4.201010E+05 rms gradient=      38.5785
 X-ray contribution to function value = 3.767700E+05
 Geometry contribution (inc ncs)      =      43331.0
 Geometry function summary
     term                           number     weight       rms        GooF      function     fn/numb
     ================================================================================================
     BOND bond lengths (angs)       5655       2.000      0.0079       0.394      1755.28       0.310
     ANGL bond angles (degs)        7677       2.000        1.01       0.600      5533.87       0.721
     TORS split-harmonic tors       1267       0.000       18.91       1.191         0.00       0.000
     SINTOR sinusoidal tors         1267       2.000       18.91                  2650.02       2.092
     PLAN general plane (angs)       962       5.000      0.0144       0.718      2481.84       2.580
     BCORrelations (angs^2)         5644      10.000       1.045       0.373      7839.83       1.389
     CONT (bad contacts)               0       5.000                                 0.00
     IDEAL(ideal-dist contact)      5063       4.000                             12442.95
     CHIRAL (gelly semiharmon)       743       5.000                                38.89
     SIM similarity restraint for NCS and/or TARGET                              10588.30
     OCCSUM restraint on sum of occupanies                                           0.00       as none defined
     DISTAN utility restraints interatomic distance                                  0.00       as none defined
     UTILANGLE utility angle restraints                                              0.00       as none defined
     UTILTOR utility restraints on torsion angles                                    0.00       as none defined
     Peptide omega torsion angles                           2.89 degs - monitor only

 Weighted rms      0.0079         1.007  (temporary rms bond and angle for BUSTER to pick up!)
 Weighted intra-residual rms(bond) for StdProtein (excluding H)      0.0077
 Weighted intra-residual rms(bond) for Buffer     (excluding H)      0.0027

 ############### Running initial check


 Initial Geometry Sanity Check

 Check for duplicate bonds: no duplicates found
 
 Check for duplicate or missing bond angles: all are OK
 Check for bond between an atom and itself not involving symmetry:  all are OK
   There are no bad contacts where Delta/sigma is greater than     6.5
   Worst contact dist=   2.345 std=   2.930 sigma=   0.101 abs(Delta/sigma)=    5.794 ats A|28:N.B (ASN) W|274:O (HOH)
   It is likely that the geometry libraries, sequence info and links match the input molecule
 GEOMETRY CHECK OVERALL:  PASS


 ############### Creating geometry/screen report


 ############### Option "tao" - run Tao optimizer

 TAO: Toolkit for Advanced Optimization. Reference: 
     Munson, T., Sarich, J., Wild, S., Benson, S., McInnes, L.C. (2018)
     "TAO User Manual (Revision 3.9)", Mathematics and Computer Science
     Division, Argonne National Laboratory
     ANL/MCS-TM-322, http://www.mcs.anl.gov/petsc/
 
 Have found -keeppdb flag so will write out a .pdb file every 1000000 its during opt
 Will apply TAO optimization 
 Maximum number of iterations      100
 Convergence test on Grms       4.0000 (-glim)
 Limit on rms displacement to original position (rmsD2init): NONE test inactive (-dlim)

    analysing argument no 0 : -autoncs with j=0
    analysing argument no 1 : -hydrogens_input_occ2zero with j=0
    analysing argument no 2 : -hydrogens_output_occ2heavy with j=0
    analysing argument no 3 : -screen with j=0
    analysing argument no 4 : 100 with j=0
    analysing argument no 5 : -screen_sigma with j=0
    analysing argument no 6 : 3.0 with j=0
    analysing argument no 7 : -keeppdb with j=0
    analysing argument no 8 : 1000000 with j=0
    analysing argument no 9 : -taomonitor_verbose with j=0
    found argument starting with "-tao" : -taomonitor_verbose
    analysing argument no 10 : 2 with j=1
    found value after -tao/-options_/-viewer_ argument : "2"
 PETSC/TAO: setting option "-taomonitor_verbose" = "2"


   MaxLik Scaling Cycle 0003
   Iters     Tot_Funct    Xray_Funct    Geom_Funct          Grms     rmsD2init     maxD2init             R         Rfree           LLG       LLGfree     rms(bond)    rms(angle)       Opttag_H
   Iters             K   B_IMPF_FRAG        K_MISS   B_TEMP_MISS   K_IMPF_MISS   B_IMPF_MISS        K_SOLV   B_TEMP_SOLV   K_IMPF_SOLV   B_IMPF_SOLV        B_TEMP     B_TEMP_11     B_TEMP_12     B_TEMP_22     B_TEMP_13     B_TEMP_23     B_TEMP_33      K_WILSON      B_WILSON      FGCYCLES       Scatag_H
   Iters          BOND         ANGLE      IMPROPER       TORSION    SINTORSION      TRIGONAL        PLANAR  BCORRELATION       CONTACT     IDEALDIST        CHIRAL    SIMILARITY       OCCSSUM  UTILDISTANCE     UTILANGLE   UTILTORSION        RHOFIT        ADP-01        ADP-02        ADP-03        ADP-04        ADP-05        ADP-06        ADP-07        ADP-08        ADP-09        ADP-10      RHOFITPC            QM       Funtag_H
       0  4.201005E+05  3.767695E+05       43331.0       38.5002  0.000000E+00  0.000000E+00      0.191801      0.222521  0.000000E+00  0.000000E+00    0.00790834       1.00733       Opttag_D
       0       86.0126       1.38271       1.00000  0.000000E+00       1.00000  0.000000E+00      0.937117       68.8243       1.00000       27.0752  0.000000E+00      -2.47894  0.000000E+00      -2.47894  0.000000E+00  0.000000E+00       4.95789       86.8527     0.0103683        2       Scatag_D
       0       1755.28       5533.87  0.000000E+00  0.000000E+00       2650.02  0.000000E+00       2481.84       7839.84  0.000000E+00       12442.9       38.8899       10588.3 -1.000000E+10 -1.000000E+10 -1.000000E+10 -1.000000E+10 -1.000000E+10  0.000000E+00  0.000000E+00  0.000000E+00  0.000000E+00  0.000000E+00  0.000000E+00  0.000000E+00  0.000000E+00  0.000000E+00  0.000000E+00  0.000000E+00  0.000000E+00       Funtag_D
   Geometry Restraint Deviations SCREEN Information

 

 WARNING: have displacement of 18.88 Angs from initial (> 15.00 Angs)
          for variable number= 24736 (type Z        ; returned function value as   1.792281E+010 )
          Involving atom W|274:O

 An atom has moved more than     1.50 A from the initial/last
 position - if this was an accepted trial point, probably need to update the
 non-bonded contact list.


   MaxLik Scaling Cycle 0004

   MaxLik Scaling Cycle 0005

   Wilson plot

   Statistics in resolution bins after refinement cycle 0001
```


|  |  |
| --- | --- |
|  | - Rxpct factors plot- Rxpct factors table- <Log-Lik Gain>- Ln Sigma A- Correlation Coefficients |


```
   Iters     Tot_Funct    Xray_Funct    Geom_Funct          Grms     rmsD2init     maxD2init             R         Rfree           LLG       LLGfree     rms(bond)    rms(angle)
   Iters             K   B_IMPF_FRAG        K_MISS   B_TEMP_MISS   K_IMPF_MISS   B_IMPF_MISS        K_SOLV   B_TEMP_SOLV   K_IMPF_SOLV   B_IMPF_SOLV        B_TEMP     B_TEMP_11     B_TEMP_12     B_TEMP_22     B_TEMP_13     B_TEMP_23     B_TEMP_33      K_WILSON      B_WILSON      FGCYCLES
   Iters          BOND         ANGLE      IMPROPER       TORSION    SINTORSION      TRIGONAL        PLANAR  BCORRELATION       CONTACT     IDEALDIST        CHIRAL    SIMILARITY       OCCSSUM  UTILDISTANCE     UTILANGLE   UTILTORSION        RHOFIT        ADP-01        ADP-02        ADP-03        ADP-04        ADP-05        ADP-06        ADP-07        ADP-08        ADP-09        ADP-10      RHOFITPC            QM
       1  4.197488E+05  3.763660E+05       43382.8       53.5331    0.00110769     0.0225464      0.191648      0.222469  9.187481E-04  2.788663E-04    0.00796596       1.00993       Opttag_D
       1       85.9756       1.36776       1.00000  0.000000E+00       1.00000  0.000000E+00      0.937189       68.6370       1.00000       27.0469  0.000000E+00      -2.46428  0.000000E+00      -2.46428  0.000000E+00  0.000000E+00       4.92856       86.8527     0.0103683        2       Scatag_D
       1       1780.96       5562.51  0.000000E+00  0.000000E+00       2649.91  0.000000E+00       2478.37       7917.99  0.000000E+00       12369.4       40.3270       10583.4 -1.000000E+10 -1.000000E+10 -1.000000E+10 -1.000000E+10 -1.000000E+10  0.000000E+00  0.000000E+00  0.000000E+00  0.000000E+00  0.000000E+00  0.000000E+00  0.000000E+00  0.000000E+00  0.000000E+00  0.000000E+00  0.000000E+00  0.000000E+00       Funtag_D
   Geometry Restraint Deviations SCREEN Information

 

   MaxLik Scaling Cycle 0006

   Wilson plot

   Statistics in resolution bins after refinement cycle 0002
```


|  |  |
| --- | --- |
|  | - Rxpct factors plot- Rxpct factors table- <Log-Lik Gain>- Ln Sigma A- Correlation Coefficients |


```
   Iters     Tot_Funct    Xray_Funct    Geom_Funct          Grms     rmsD2init     maxD2init             R         Rfree           LLG       LLGfree     rms(bond)    rms(angle)
   Iters             K   B_IMPF_FRAG        K_MISS   B_TEMP_MISS   K_IMPF_MISS   B_IMPF_MISS        K_SOLV   B_TEMP_SOLV   K_IMPF_SOLV   B_IMPF_SOLV        B_TEMP     B_TEMP_11     B_TEMP_12     B_TEMP_22     B_TEMP_13     B_TEMP_23     B_TEMP_33      K_WILSON      B_WILSON      FGCYCLES
   Iters          BOND         ANGLE      IMPROPER       TORSION    SINTORSION      TRIGONAL        PLANAR  BCORRELATION       CONTACT     IDEALDIST        CHIRAL    SIMILARITY       OCCSSUM  UTILDISTANCE     UTILANGLE   UTILTORSION        RHOFIT        ADP-01        ADP-02        ADP-03        ADP-04        ADP-05        ADP-06        ADP-07        ADP-08        ADP-09        ADP-10      RHOFITPC            QM
       2  4.193626E+05  3.761659E+05       43196.8       35.8172    0.00193696     0.0424169      0.191561      0.222478    0.00137816  1.822132E-04    0.00801138       1.01076       Opttag_D
       2       85.9514       1.36043       1.00000  0.000000E+00       1.00000  0.000000E+00      0.937191       68.5722       1.00000       27.0033  0.000000E+00      -2.45856  0.000000E+00      -2.45856  0.000000E+00  0.000000E+00       4.91713       86.8527     0.0103683        1       Scatag_D
       2       1801.32       5571.67  0.000000E+00  0.000000E+00       2649.95  0.000000E+00       2476.89       7756.34  0.000000E+00       12313.7       40.8564       10586.0 -1.000000E+10 -1.000000E+10 -1.000000E+10 -1.000000E+10 -1.000000E+10  0.000000E+00  0.000000E+00  0.000000E+00  0.000000E+00  0.000000E+00  0.000000E+00  0.000000E+00  0.000000E+00  0.000000E+00  0.000000E+00  0.000000E+00  0.000000E+00       Funtag_D
   Geometry Restraint Deviations SCREEN Information

 
       3  4.190691E+05  3.759162E+05       43152.9       34.5831    0.00308702     0.0703629      0.191449      0.222478    0.00195141  7.118758E-05    0.00807783       1.01313       Opttag_D
       3       85.9205       1.35143       1.00000  0.000000E+00       1.00000  0.000000E+00      0.937194       68.4943       1.00000       26.9475  0.000000E+00      -2.45222  0.000000E+00      -2.45222  0.000000E+00  0.000000E+00       4.90445       86.8527     0.0103683        1       Scatag_D
       3       1831.33       5597.81  0.000000E+00  0.000000E+00       2650.01  0.000000E+00       2476.95       7717.37  0.000000E+00       12242.4       41.2740       10595.8 -1.000000E+10 -1.000000E+10 -1.000000E+10 -1.000000E+10 -1.000000E+10  0.000000E+00  0.000000E+00  0.000000E+00  0.000000E+00  0.000000E+00  0.000000E+00  0.000000E+00  0.000000E+00  0.000000E+00  0.000000E+00  0.000000E+00  0.000000E+00       Funtag_D
       4  4.187219E+05  3.753984E+05       43323.5       51.1482    0.00568442      0.134037      0.191218      0.222465    0.00314019 -1.289672E-04    0.00826497       1.02134       Opttag_D
       4       85.8559       1.33288       1.00000  0.000000E+00       1.00000  0.000000E+00      0.937224       68.3201       1.00000       26.8340  0.000000E+00      -2.43770  0.000000E+00      -2.43770  0.000000E+00  0.000000E+00       4.87539       86.8527     0.0103683        1       Scatag_D
       4       1917.17       5688.86  0.000000E+00  0.000000E+00       2650.13  0.000000E+00       2481.46       7803.73  0.000000E+00       12101.1       42.1345       10638.9 -1.000000E+10 -1.000000E+10 -1.000000E+10 -1.000000E+10 -1.000000E+10  0.000000E+00  0.000000E+00  0.000000E+00  0.000000E+00  0.000000E+00  0.000000E+00  0.000000E+00  0.000000E+00  0.000000E+00  0.000000E+00  0.000000E+00  0.000000E+00       Funtag_D
       5  4.183793E+05  3.749882E+05       43391.1       41.2531    0.00805682      0.193951      0.191035      0.222447    0.00408181 -2.880300E-04    0.00839567       1.02351       Opttag_D
       5       85.8018       1.31821       1.00000  0.000000E+00       1.00000  0.000000E+00      0.937276       68.1710       1.00000       26.7387  0.000000E+00      -2.42413  0.000000E+00      -2.42413  0.000000E+00  0.000000E+00       4.84826       86.8527     0.0103683        1       Scatag_D
       5       1978.28       5713.10  0.000000E+00  0.000000E+00       2650.31  0.000000E+00       2489.84       7836.28  0.000000E+00       11990.9       42.6991       10689.7 -1.000000E+10 -1.000000E+10 -1.000000E+10 -1.000000E+10 -1.000000E+10  0.000000E+00  0.000000E+00  0.000000E+00  0.000000E+00  0.000000E+00  0.000000E+00  0.000000E+00  0.000000E+00  0.000000E+00  0.000000E+00  0.000000E+00  0.000000E+00       Funtag_D
       6  4.180733E+05  3.747532E+05       43320.1       33.9912    0.00980571      0.239718      0.190925      0.222438    0.00462121 -3.867087E-04    0.00843245       1.02588       Opttag_D
       6       85.7659       1.30992       1.00000  0.000000E+00       1.00000  0.000000E+00      0.937321       68.0882       1.00000       26.6705  0.000000E+00      -2.41563  0.000000E+00      -2.41563  0.000000E+00  0.000000E+00       4.83125       86.8527     0.0103683        1       Scatag_D
       6       1995.65       5739.56  0.000000E+00  0.000000E+00       2650.51  0.000000E+00       2498.47       7758.51  0.000000E+00       11918.3       42.6726       10716.4 -1.000000E+10 -1.000000E+10 -1.000000E+10 -1.000000E+10 -1.000000E+10  0.000000E+00  0.000000E+00  0.000000E+00  0.000000E+00  0.000000E+00  0.000000E+00  0.000000E+00  0.000000E+00  0.000000E+00  0.000000E+00  0.000000E+00  0.000000E+00       Funtag_D
       7  4.176998E+05  3.744255E+05       43274.3       42.5705     0.0130842      0.325272      0.190760      0.222427    0.00537362 -6.232290E-04    0.00844829       1.03165       Opttag_D
       7       85.7065       1.29825       1.00000  0.000000E+00       1.00000  0.000000E+00      0.937414       67.9670       1.00000       26.5509  0.000000E+00      -2.40034  0.000000E+00      -2.40034  0.000000E+00  0.000000E+00       4.80068       86.8527     0.0103683        1       Scatag_D
       7       2003.16       5804.28  0.000000E+00  0.000000E+00       2650.72  0.000000E+00       2516.47       7700.71  0.000000E+00       11805.9       42.5610       10750.5 -1.000000E+10 -1.000000E+10 -1.000000E+10 -1.000000E+10 -1.000000E+10  0.000000E+00  0.000000E+00  0.000000E+00  0.000000E+00  0.000000E+00  0.000000E+00  0.000000E+00  0.000000E+00  0.000000E+00  0.000000E+00  0.000000E+00  0.000000E+00       Funtag_D
       8  4.174358E+05  3.741813E+05       43254.5       48.5145     0.0165462      0.413060      0.190644      0.222428    0.00593421 -9.834112E-04    0.00845571       1.03779       Opttag_D
       8       85.6514       1.28898       1.00000  0.000000E+00       1.00000  0.000000E+00      0.937517       67.8557       1.00000       26.4361  0.000000E+00      -2.38285  0.000000E+00      -2.38285  0.000000E+00  0.000000E+00       4.76570       86.8527     0.0103683        1       Scatag_D
       8       2006.67       5873.55  0.000000E+00  0.000000E+00       2651.23  0.000000E+00       2534.82       7651.46  0.000000E+00       11716.7       42.7184       10777.3 -1.000000E+10 -1.000000E+10 -1.000000E+10 -1.000000E+10 -1.000000E+10  0.000000E+00  0.000000E+00  0.000000E+00  0.000000E+00  0.000000E+00  0.000000E+00  0.000000E+00  0.000000E+00  0.000000E+00  0.000000E+00  0.000000E+00  0.000000E+00       Funtag_D
       9  4.171814E+05  3.741550E+05       43026.5       30.0538     0.0174523      0.434904      0.190627      0.222432    0.00599469   -0.00111595    0.00840263       1.03335       Opttag_D
       9       85.6389       1.28794       1.00000  0.000000E+00       1.00000  0.000000E+00      0.937546       67.8465       1.00000       26.4050  0.000000E+00      -2.37860  0.000000E+00      -2.37860  0.000000E+00  0.000000E+00       4.75720       86.8527     0.0103683        1       Scatag_D
       9       1981.56       5823.44  0.000000E+00  0.000000E+00       2651.11  0.000000E+00       2536.74       7532.76  0.000000E+00       11696.5       42.7154       10761.6 -1.000000E+10 -1.000000E+10 -1.000000E+10 -1.000000E+10 -1.000000E+10  0.000000E+00  0.000000E+00  0.000000E+00  0.000000E+00  0.000000E+00  0.000000E+00  0.000000E+00  0.000000E+00  0.000000E+00  0.000000E+00  0.000000E+00  0.000000E+00       Funtag_D
      10  4.169024E+05  3.740799E+05       42822.4       26.4315     0.0194392      0.479505      0.190588      0.222459    0.00616698   -0.00143919    0.00835223       1.03262       Opttag_D
      10       85.6120       1.28482       1.00000  0.000000E+00       1.00000  0.000000E+00      0.937619       67.8076       1.00000       26.3434  0.000000E+00      -2.36821  0.000000E+00      -2.36821  0.000000E+00  0.000000E+00       4.73642       86.8527     0.0103683        1       Scatag_D
      10       1957.86       5815.21  0.000000E+00  0.000000E+00       2650.88  0.000000E+00       2539.38       7420.04  0.000000E+00       11658.0       42.8651       10738.1 -1.000000E+10 -1.000000E+10 -1.000000E+10 -1.000000E+10 -1.000000E+10  0.000000E+00  0.000000E+00  0.000000E+00  0.000000E+00  0.000000E+00  0.000000E+00  0.000000E+00  0.000000E+00  0.000000E+00  0.000000E+00  0.000000E+00  0.000000E+00       Funtag_D
      11  4.167150E+05  3.739723E+05       42742.7       33.0188     0.0218099      0.529115      0.190536      0.222499    0.00641398   -0.00179551    0.00834552       1.03493       Opttag_D
      11       85.5814       1.28026       1.00000  0.000000E+00       1.00000  0.000000E+00      0.937717       67.7378       1.00000       26.2794  0.000000E+00      -2.35507  0.000000E+00      -2.35507  0.000000E+00  0.000000E+00       4.71014       86.8527     0.0103683        1       Scatag_D
      11       1954.72       5841.25  0.000000E+00  0.000000E+00       2650.77  0.000000E+00       2542.91       7362.19  0.000000E+00       11620.4       42.9646       10727.4 -1.000000E+10 -1.000000E+10 -1.000000E+10 -1.000000E+10 -1.000000E+10  0.000000E+00  0.000000E+00  0.000000E+00  0.000000E+00  0.000000E+00  0.000000E+00  0.000000E+00  0.000000E+00  0.000000E+00  0.000000E+00  0.000000E+00  0.000000E+00       Funtag_D
      12  4.164926E+05  3.738358E+05       42656.8       28.2459     0.0252650      0.594408      0.190466      0.222587    0.00672741   -0.00232344    0.00835222       1.03505       Opttag_D
      12       85.5413       1.27412       1.00000  0.000000E+00       1.00000  0.000000E+00      0.937875       67.6303       1.00000       26.1965  0.000000E+00      -2.33624  0.000000E+00      -2.33624  0.000000E+00  0.000000E+00       4.67248       86.8527     0.0103683        1       Scatag_D
      12       1957.86       5842.59  0.000000E+00  0.000000E+00       2650.58  0.000000E+00       2546.06       7323.51  0.000000E+00       11576.0       43.0697       10717.1 -1.000000E+10 -1.000000E+10 -1.000000E+10 -1.000000E+10 -1.000000E+10  0.000000E+00  0.000000E+00  0.000000E+00  0.000000E+00  0.000000E+00  0.000000E+00  0.000000E+00  0.000000E+00  0.000000E+00  0.000000E+00  0.000000E+00  0.000000E+00       Funtag_D
      13  4.163279E+05  3.737340E+05       42593.9       29.4964     0.0275312      0.630283      0.190415      0.222650    0.00696109   -0.00262159    0.00835578       1.03706       Opttag_D
      13       85.5177       1.26974       1.00000  0.000000E+00       1.00000  0.000000E+00      0.937988       67.5546       1.00000       26.1458  0.000000E+00      -2.32558  0.000000E+00      -2.32558  0.000000E+00  0.000000E+00       4.65117       86.8527     0.0103683        1       Scatag_D
      13       1959.52       5865.39  0.000000E+00  0.000000E+00       2650.67  0.000000E+00       2545.83       7269.18  0.000000E+00       11549.5       43.0808       10710.7 -1.000000E+10 -1.000000E+10 -1.000000E+10 -1.000000E+10 -1.000000E+10  0.000000E+00  0.000000E+00  0.000000E+00  0.000000E+00  0.000000E+00  0.000000E+00  0.000000E+00  0.000000E+00  0.000000E+00  0.000000E+00  0.000000E+00  0.000000E+00       Funtag_D
      14  4.161843E+05  3.736588E+05       42525.5       21.9032     0.0286878      0.642576      0.190376      0.222685    0.00713372   -0.00274294    0.00834697       1.03606       Opttag_D
      14       85.5070       1.26698       1.00000  0.000000E+00       1.00000  0.000000E+00      0.938052       67.5151       1.00000       26.1186  0.000000E+00      -2.32177  0.000000E+00      -2.32177  0.000000E+00  0.000000E+00       4.64353       86.8527     0.0103683        1       Scatag_D
      14       1955.40       5853.97  0.000000E+00  0.000000E+00       2650.66  0.000000E+00       2543.12       7243.38  0.000000E+00       11531.7       43.1653       10704.1 -1.000000E+10 -1.000000E+10 -1.000000E+10 -1.000000E+10 -1.000000E+10  0.000000E+00  0.000000E+00  0.000000E+00  0.000000E+00  0.000000E+00  0.000000E+00  0.000000E+00  0.000000E+00  0.000000E+00  0.000000E+00  0.000000E+00  0.000000E+00       Funtag_D
      15  4.160507E+05  3.735555E+05       42495.2       22.4181     0.0302597      0.654990      0.190319      0.222735    0.00737084   -0.00292409    0.00834929       1.03621       Opttag_D
      15       85.4951       1.26347       1.00000  0.000000E+00       1.00000  0.000000E+00      0.938134       67.4712       1.00000       26.0815  0.000000E+00      -2.31830  0.000000E+00      -2.31830  0.000000E+00  0.000000E+00       4.63659       86.8527     0.0103683        1       Scatag_D
      15       1956.48       5855.71  0.000000E+00  0.000000E+00       2650.74  0.000000E+00       2540.93       7238.41  0.000000E+00       11509.1       43.4813       10700.3 -1.000000E+10 -1.000000E+10 -1.000000E+10 -1.000000E+10 -1.000000E+10  0.000000E+00  0.000000E+00  0.000000E+00  0.000000E+00  0.000000E+00  0.000000E+00  0.000000E+00  0.000000E+00  0.000000E+00  0.000000E+00  0.000000E+00  0.000000E+00       Funtag_D
      16  4.159371E+05  3.734474E+05       42489.7       24.4125     0.0320809      0.666349      0.190263      0.222793    0.00761919   -0.00321270    0.00835974       1.03766       Opttag_D
      16       85.4843       1.25995       1.00000  0.000000E+00       1.00000  0.000000E+00      0.938221       67.4313       1.00000       26.0391  0.000000E+00      -2.31521  0.000000E+00      -2.31521  0.000000E+00  0.000000E+00       4.63042       86.8527     0.0103683        1       Scatag_D
      16       1961.39       5872.15  0.000000E+00  0.000000E+00       2651.04  0.000000E+00       2541.58       7230.90  0.000000E+00       11487.0       43.9102       10701.7 -1.000000E+10 -1.000000E+10 -1.000000E+10 -1.000000E+10 -1.000000E+10  0.000000E+00  0.000000E+00  0.000000E+00  0.000000E+00  0.000000E+00  0.000000E+00  0.000000E+00  0.000000E+00  0.000000E+00  0.000000E+00  0.000000E+00  0.000000E+00       Funtag_D
      17  4.158277E+05  3.733748E+05       42452.9       18.3245     0.0335009      0.675665      0.190223      0.222829    0.00778578   -0.00348617    0.00836160       1.03719       Opttag_D
      17       85.4768       1.25759       1.00000  0.000000E+00       1.00000  0.000000E+00      0.938284       67.4046       1.00000       26.0073  0.000000E+00      -2.31214  0.000000E+00      -2.31214  0.000000E+00  0.000000E+00       4.62428       86.8527     0.0103683        1       Scatag_D
      17       1962.25       5866.77  0.000000E+00  0.000000E+00       2651.39  0.000000E+00       2544.57       7206.49  0.000000E+00       11474.6       44.0663       10702.8 -1.000000E+10 -1.000000E+10 -1.000000E+10 -1.000000E+10 -1.000000E+10  0.000000E+00  0.000000E+00  0.000000E+00  0.000000E+00  0.000000E+00  0.000000E+00  0.000000E+00  0.000000E+00  0.000000E+00  0.000000E+00  0.000000E+00  0.000000E+00       Funtag_D
      18  4.157062E+05  3.732626E+05       42443.6       19.3930     0.0357906      0.689281      0.190160      0.222896    0.00804325   -0.00394618    0.00836567       1.03771       Opttag_D
      18       85.4662       1.25398       1.00000  0.000000E+00       1.00000  0.000000E+00      0.938388       67.3629       1.00000       25.9578  0.000000E+00      -2.30674  0.000000E+00      -2.30674  0.000000E+00  0.000000E+00       4.61348       86.8527     0.0103683        1       Scatag_D
      18       1964.17       5872.67  0.000000E+00  0.000000E+00       2651.95  0.000000E+00       2551.49       7196.28  0.000000E+00       11458.7       44.1030       10704.2 -1.000000E+10 -1.000000E+10 -1.000000E+10 -1.000000E+10 -1.000000E+10  0.000000E+00  0.000000E+00  0.000000E+00  0.000000E+00  0.000000E+00  0.000000E+00  0.000000E+00  0.000000E+00  0.000000E+00  0.000000E+00  0.000000E+00  0.000000E+00       Funtag_D
      19  4.156225E+05  3.731655E+05       42457.0       23.3002     0.0378874      0.699219      0.190101      0.222945    0.00826614   -0.00433087    0.00836610       1.03888       Opttag_D
      19       85.4583       1.25087       1.00000  0.000000E+00       1.00000  0.000000E+00      0.938489       67.3254       1.00000       25.9133  0.000000E+00      -2.30192  0.000000E+00      -2.30192  0.000000E+00  0.000000E+00       4.60384       86.8527     0.0103683        1       Scatag_D
      19       1964.37       5885.93  0.000000E+00  0.000000E+00       2652.60  0.000000E+00       2558.53       7196.06  0.000000E+00       11447.8       43.9887       10707.7 -1.000000E+10 -1.000000E+10 -1.000000E+10 -1.000000E+10 -1.000000E+10  0.000000E+00  0.000000E+00  0.000000E+00  0.000000E+00  0.000000E+00  0.000000E+00  0.000000E+00  0.000000E+00  0.000000E+00  0.000000E+00  0.000000E+00  0.000000E+00       Funtag_D
      20  4.155375E+05  3.730950E+05       42442.4       16.2338     0.0393519      0.703623      0.190061      0.222984    0.00842804   -0.00459710    0.00835749       1.03820       Opttag_D
      20       85.4538       1.24871       1.00000  0.000000E+00       1.00000  0.000000E+00      0.938560       67.3025       1.00000       25.8808  0.000000E+00      -2.29874  0.000000E+00      -2.29874  0.000000E+00  0.000000E+00       4.59748       86.8527     0.0103683        1       Scatag_D
      20       1960.33       5878.28  0.000000E+00  0.000000E+00       2652.94  0.000000E+00       2563.63       7192.56  0.000000E+00       11440.6       43.7081       10710.3 -1.000000E+10 -1.000000E+10 -1.000000E+10 -1.000000E+10 -1.000000E+10  0.000000E+00  0.000000E+00  0.000000E+00  0.000000E+00  0.000000E+00  0.000000E+00  0.000000E+00  0.000000E+00  0.000000E+00  0.000000E+00  0.000000E+00  0.000000E+00       Funtag_D
      21  4.154381E+05  3.729895E+05       42448.6       15.9509     0.0414756      0.705557      0.189997      0.223023    0.00867019   -0.00494378    0.00834509       1.03849       Opttag_D
      21       85.4495       1.24568       1.00000  0.000000E+00       1.00000  0.000000E+00      0.938661       67.2763       1.00000       25.8321  0.000000E+00      -2.29554  0.000000E+00      -2.29554  0.000000E+00  0.000000E+00       4.59109       86.8527     0.0103683        1       Scatag_D
      21       1954.52       5881.47  0.000000E+00  0.000000E+00       2653.40  0.000000E+00       2571.26       7196.23  0.000000E+00       11431.5       43.1163       10717.1 -1.000000E+10 -1.000000E+10 -1.000000E+10 -1.000000E+10 -1.000000E+10  0.000000E+00  0.000000E+00  0.000000E+00  0.000000E+00  0.000000E+00  0.000000E+00  0.000000E+00  0.000000E+00  0.000000E+00  0.000000E+00  0.000000E+00  0.000000E+00       Funtag_D
      22  4.153684E+05  3.729039E+05       42464.6       19.4001     0.0431334      0.704021      0.189944      0.223075    0.00886694   -0.00527116    0.00834208       1.03968       Opttag_D
      22       85.4482       1.24336       1.00000  0.000000E+00       1.00000  0.000000E+00      0.938729       67.2623       1.00000       25.7938  0.000000E+00      -2.29422  0.000000E+00      -2.29422  0.000000E+00  0.000000E+00       4.58845       86.8527     0.0103683        1       Scatag_D
      22       1953.10       5894.99  0.000000E+00  0.000000E+00       2653.69  0.000000E+00       2577.11       7194.80  0.000000E+00       11423.7       42.8637       10724.4 -1.000000E+10 -1.000000E+10 -1.000000E+10 -1.000000E+10 -1.000000E+10  0.000000E+00  0.000000E+00  0.000000E+00  0.000000E+00  0.000000E+00  0.000000E+00  0.000000E+00  0.000000E+00  0.000000E+00  0.000000E+00  0.000000E+00  0.000000E+00       Funtag_D
      23  4.152949E+05  3.728272E+05       42467.7       15.1558     0.0445833      0.700468      0.189900      0.223120    0.00904291   -0.00557597    0.00834238       1.03959       Opttag_D
      23       85.4491       1.24142       1.00000  0.000000E+00       1.00000  0.000000E+00      0.938783       67.2549       1.00000       25.7609  0.000000E+00      -2.29390  0.000000E+00      -2.29390  0.000000E+00  0.000000E+00       4.58780       86.8527     0.0103683        1       Scatag_D
      23       1953.25       5894.02  0.000000E+00  0.000000E+00       2653.93  0.000000E+00       2582.38       7192.08  0.000000E+00       11417.0       42.8302       10732.2 -1.000000E+10 -1.000000E+10 -1.000000E+10 -1.000000E+10 -1.000000E+10  0.000000E+00  0.000000E+00  0.000000E+00  0.000000E+00  0.000000E+00  0.000000E+00  0.000000E+00  0.000000E+00  0.000000E+00  0.000000E+00  0.000000E+00  0.000000E+00       Funtag_D
      24  4.152185E+05  3.727251E+05       42493.4       14.1298     0.0464620      0.693401      0.189840      0.223179    0.00927719   -0.00601902    0.00835420       1.04018       Opttag_D
      24       85.4538       1.23909       1.00000  0.000000E+00       1.00000  0.000000E+00      0.938846       67.2508       1.00000       25.7209  0.000000E+00      -2.29440  0.000000E+00      -2.29440  0.000000E+00  0.000000E+00       4.58880       86.8527     0.0103683        1       Scatag_D
      24       1958.78       5900.63  0.000000E+00  0.000000E+00       2654.35  0.000000E+00       2589.53       7194.91  0.000000E+00       11409.2       43.1907       10742.8 -1.000000E+10 -1.000000E+10 -1.000000E+10 -1.000000E+10 -1.000000E+10  0.000000E+00  0.000000E+00  0.000000E+00  0.000000E+00  0.000000E+00  0.000000E+00  0.000000E+00  0.000000E+00  0.000000E+00  0.000000E+00  0.000000E+00  0.000000E+00       Funtag_D
      25  4.151636E+05  3.726423E+05       42521.3       17.9841     0.0480751      0.686220      0.189791      0.223226    0.00946740   -0.00638296    0.00836811       1.04150       Opttag_D
      25       85.4610       1.23746       1.00000  0.000000E+00       1.00000  0.000000E+00      0.938899       67.2519       1.00000       25.6895  0.000000E+00      -2.29473  0.000000E+00      -2.29473  0.000000E+00  0.000000E+00       4.58947       86.8527     0.0103683        1       Scatag_D
      25       1965.31       5915.71  0.000000E+00  0.000000E+00       2654.68  0.000000E+00       2595.79       7192.29  0.000000E+00       11403.4       43.6262       10750.6 -1.000000E+10 -1.000000E+10 -1.000000E+10 -1.000000E+10 -1.000000E+10  0.000000E+00  0.000000E+00  0.000000E+00  0.000000E+00  0.000000E+00  0.000000E+00  0.000000E+00  0.000000E+00  0.000000E+00  0.000000E+00  0.000000E+00  0.000000E+00       Funtag_D
      26  4.151073E+05  3.725892E+05       42518.1       12.7925     0.0490616      0.679427      0.189757      0.223256    0.00958919   -0.00662093    0.00837632       1.04120       Opttag_D
      26       85.4687       1.23672       1.00000  0.000000E+00       1.00000  0.000000E+00      0.938929       67.2608       1.00000       25.6707  0.000000E+00      -2.29552  0.000000E+00      -2.29552  0.000000E+00  0.000000E+00       4.59103       86.8527     0.0103683        1       Scatag_D
      26       1969.17       5912.23  0.000000E+00  0.000000E+00       2655.05  0.000000E+00       2599.23       7185.90  0.000000E+00       11399.1       44.0059       10753.4 -1.000000E+10 -1.000000E+10 -1.000000E+10 -1.000000E+10 -1.000000E+10  0.000000E+00  0.000000E+00  0.000000E+00  0.000000E+00  0.000000E+00  0.000000E+00  0.000000E+00  0.000000E+00  0.000000E+00  0.000000E+00  0.000000E+00  0.000000E+00       Funtag_D
      27  4.150424E+05  3.725219E+05       42520.5       11.9292     0.0505284      0.668265      0.189707      0.223285    0.00974389   -0.00689931    0.00838223       1.04170       Opttag_D
      27       85.4830       1.23622       1.00000  0.000000E+00       1.00000  0.000000E+00      0.938973       67.2849       1.00000       25.6437  0.000000E+00      -2.29675  0.000000E+00      -2.29675  0.000000E+00  0.000000E+00       4.59351       86.8527     0.0103683        1       Scatag_D
      27       1971.95       5917.93  0.000000E+00  0.000000E+00       2655.65  0.000000E+00       2604.19       7176.66  0.000000E+00       11394.1       44.3161       10755.7 -1.000000E+10 -1.000000E+10 -1.000000E+10 -1.000000E+10 -1.000000E+10  0.000000E+00  0.000000E+00  0.000000E+00  0.000000E+00  0.000000E+00  0.000000E+00  0.000000E+00  0.000000E+00  0.000000E+00  0.000000E+00  0.000000E+00  0.000000E+00       Funtag_D
      28  4.149951E+05  3.724512E+05       42543.9       19.6205     0.0524068      0.654139      0.189645      0.223327    0.00990613   -0.00726239    0.00839432       1.04317       Opttag_D
      28       85.5025       1.23600       1.00000  0.000000E+00       1.00000  0.000000E+00      0.939032       67.3220       1.00000       25.6104  0.000000E+00      -2.29811  0.000000E+00      -2.29811  0.000000E+00  0.000000E+00       4.59622       86.8527     0.0103683        1       Scatag_D
      28       1977.65       5934.69  0.000000E+00  0.000000E+00       2656.41  0.000000E+00       2609.95       7170.64  0.000000E+00       11390.1       44.3848       10760.0 -1.000000E+10 -1.000000E+10 -1.000000E+10 -1.000000E+10 -1.000000E+10  0.000000E+00  0.000000E+00  0.000000E+00  0.000000E+00  0.000000E+00  0.000000E+00  0.000000E+00  0.000000E+00  0.000000E+00  0.000000E+00  0.000000E+00  0.000000E+00       Funtag_D
      29  4.149422E+05  3.724163E+05       42525.9       12.0998     0.0534128      0.660575      0.189610      0.223342    0.00998629   -0.00741682    0.00838649       1.04265       Opttag_D
      29       85.5146       1.23611       1.00000  0.000000E+00       1.00000  0.000000E+00      0.939063       67.3494       1.00000       25.5929  0.000000E+00      -2.29891  0.000000E+00      -2.29891  0.000000E+00  0.000000E+00       4.59782       86.8527     0.0103683        1       Scatag_D
      29       1973.96       5928.77  0.000000E+00  0.000000E+00       2656.88  0.000000E+00       2612.01       7160.93  0.000000E+00       11388.3       44.2738       10760.8 -1.000000E+10 -1.000000E+10 -1.000000E+10 -1.000000E+10 -1.000000E+10  0.000000E+00  0.000000E+00  0.000000E+00  0.000000E+00  0.000000E+00  0.000000E+00  0.000000E+00  0.000000E+00  0.000000E+00  0.000000E+00  0.000000E+00  0.000000E+00       Funtag_D
      30  4.148878E+05  3.723686E+05       42519.2       10.5868     0.0547164      0.682988      0.189562      0.223372     0.0100957   -0.00762990    0.00838101       1.04290       Opttag_D
      30       85.5309       1.23615       1.00000  0.000000E+00       1.00000  0.000000E+00      0.939109       67.3861       1.00000       25.5704  0.000000E+00      -2.29989  0.000000E+00      -2.29989  0.000000E+00  0.000000E+00       4.59978       86.8527     0.0103683        1       Scatag_D
      30       1971.38       5931.58  0.000000E+00  0.000000E+00       2657.42  0.000000E+00       2613.39       7152.28  0.000000E+00       11387.4       44.1172       10761.7 -1.000000E+10 -1.000000E+10 -1.000000E+10 -1.000000E+10 -1.000000E+10  0.000000E+00  0.000000E+00  0.000000E+00  0.000000E+00  0.000000E+00  0.000000E+00  0.000000E+00  0.000000E+00  0.000000E+00  0.000000E+00  0.000000E+00  0.000000E+00       Funtag_D
      31  4.148434E+05  3.723033E+05       42540.2       14.3396     0.0564163      0.711223      0.189500      0.223422     0.0102458   -0.00797475    0.00838456       1.04377       Opttag_D
      31       85.5522       1.23599       1.00000  0.000000E+00       1.00000  0.000000E+00      0.939171       67.4262       1.00000       25.5440  0.000000E+00      -2.30090  0.000000E+00      -2.30090  0.000000E+00  0.000000E+00       4.60180       86.8527     0.0103683        1       Scatag_D
      31       1973.05       5941.52  0.000000E+00  0.000000E+00       2658.14  0.000000E+00       2614.97       7154.52  0.000000E+00       11388.0       44.2173       10765.8 -1.000000E+10 -1.000000E+10 -1.000000E+10 -1.000000E+10 -1.000000E+10  0.000000E+00  0.000000E+00  0.000000E+00  0.000000E+00  0.000000E+00  0.000000E+00  0.000000E+00  0.000000E+00  0.000000E+00  0.000000E+00  0.000000E+00  0.000000E+00       Funtag_D
      32  4.147972E+05  3.722356E+05       42561.6       11.8575     0.0581915      0.739642      0.189436      0.223491     0.0104010   -0.00836367    0.00839169       1.04415       Opttag_D
      32       85.5757       1.23583       1.00000  0.000000E+00       1.00000  0.000000E+00      0.939238       67.4672       1.00000       25.5190  0.000000E+00      -2.30196  0.000000E+00      -2.30196  0.000000E+00  0.000000E+00       4.60391       86.8527     0.0103683        1       Scatag_D
      32       1976.41       5945.76  0.000000E+00  0.000000E+00       2658.96  0.000000E+00       2615.80       7157.79  0.000000E+00       11391.2       44.4659       10771.2 -1.000000E+10 -1.000000E+10 -1.000000E+10 -1.000000E+10 -1.000000E+10  0.000000E+00  0.000000E+00  0.000000E+00  0.000000E+00  0.000000E+00  0.000000E+00  0.000000E+00  0.000000E+00  0.000000E+00  0.000000E+00  0.000000E+00  0.000000E+00       Funtag_D
      33  4.147589E+05  3.721889E+05       42570.0       10.0820     0.0592195      0.755332      0.189396      0.223538     0.0105083   -0.00862653    0.00839407       1.04433       Opttag_D
      33       85.5909       1.23560       1.00000  0.000000E+00       1.00000  0.000000E+00      0.939281       67.4875       1.00000       25.5063  0.000000E+00      -2.30294  0.000000E+00      -2.30294  0.000000E+00  0.000000E+00       4.60588       86.8527     0.0103683        1       Scatag_D
      33       1977.53       5947.81  0.000000E+00  0.000000E+00       2659.36  0.000000E+00       2615.35       7156.60  0.000000E+00       11394.3       44.8065       10774.2 -1.000000E+10 -1.000000E+10 -1.000000E+10 -1.000000E+10 -1.000000E+10  0.000000E+00  0.000000E+00  0.000000E+00  0.000000E+00  0.000000E+00  0.000000E+00  0.000000E+00  0.000000E+00  0.000000E+00  0.000000E+00  0.000000E+00  0.000000E+00       Funtag_D
      34  4.147171E+05  3.721333E+05       42583.8       11.0342     0.0604224      0.772763      0.189347      0.223598     0.0106359   -0.00895980    0.00839798       1.04473       Opttag_D
      34       85.6109       1.23543       1.00000  0.000000E+00       1.00000  0.000000E+00      0.939331       67.5107       1.00000       25.4931  0.000000E+00      -2.30438  0.000000E+00      -2.30438  0.000000E+00  0.000000E+00       4.60877       86.8527     0.0103683        1       Scatag_D
      34       1979.37       5952.43  0.000000E+00  0.000000E+00       2659.89  0.000000E+00       2614.36       7155.55  0.000000E+00       11398.3       45.3670       10778.6 -1.000000E+10 -1.000000E+10 -1.000000E+10 -1.000000E+10 -1.000000E+10  0.000000E+00  0.000000E+00  0.000000E+00  0.000000E+00  0.000000E+00  0.000000E+00  0.000000E+00  0.000000E+00  0.000000E+00  0.000000E+00  0.000000E+00  0.000000E+00       Funtag_D
      35  4.146852E+05  3.720805E+05       42604.7       13.0967     0.0616642      0.789705      0.189303      0.223652     0.0107571   -0.00928304    0.00839952       1.04520       Opttag_D
      35       85.6334       1.23548       1.00000  0.000000E+00       1.00000  0.000000E+00      0.939379       67.5375       1.00000       25.4811  0.000000E+00      -2.30619  0.000000E+00      -2.30619  0.000000E+00  0.000000E+00       4.61238       86.8527     0.0103683        1       Scatag_D
      35       1980.10       5957.74  0.000000E+00  0.000000E+00       2660.49  0.000000E+00       2614.11       7159.54  0.000000E+00       11403.1       45.8555       10783.8 -1.000000E+10 -1.000000E+10 -1.000000E+10 -1.000000E+10 -1.000000E+10  0.000000E+00  0.000000E+00  0.000000E+00  0.000000E+00  0.000000E+00  0.000000E+00  0.000000E+00  0.000000E+00  0.000000E+00  0.000000E+00  0.000000E+00  0.000000E+00       Funtag_D
      36  4.146519E+05  3.720543E+05       42597.6       9.29815     0.0622318      0.796501      0.189277      0.223675     0.0108173   -0.00942888    0.00839685       1.04497       Opttag_D
      36       85.6465       1.23560       1.00000  0.000000E+00       1.00000  0.000000E+00      0.939400       67.5517       1.00000       25.4760  0.000000E+00      -2.30718  0.000000E+00      -2.30718  0.000000E+00  0.000000E+00       4.61437       86.8527     0.0103683        1       Scatag_D
      36       1978.84       5955.14  0.000000E+00  0.000000E+00       2660.69  0.000000E+00       2614.13       7151.88  0.000000E+00       11405.3       45.9927       10785.6 -1.000000E+10 -1.000000E+10 -1.000000E+10 -1.000000E+10 -1.000000E+10  0.000000E+00  0.000000E+00  0.000000E+00  0.000000E+00  0.000000E+00  0.000000E+00  0.000000E+00  0.000000E+00  0.000000E+00  0.000000E+00  0.000000E+00  0.000000E+00       Funtag_D
      37  4.146110E+05  3.720120E+05       42599.0       9.05273     0.0632711      0.808458      0.189240      0.223714     0.0109145   -0.00968595    0.00839521       1.04501       Opttag_D
      37       85.6702       1.23595       1.00000  0.000000E+00       1.00000  0.000000E+00      0.939434       67.5799       1.00000       25.4663  0.000000E+00      -2.30854  0.000000E+00      -2.30854  0.000000E+00  0.000000E+00       4.61708       86.8527     0.0103683        1       Scatag_D
      37       1978.07       5955.64  0.000000E+00  0.000000E+00       2661.14  0.000000E+00       2614.90       7145.85  0.000000E+00       11408.9       46.0203       10788.5 -1.000000E+10 -1.000000E+10 -1.000000E+10 -1.000000E+10 -1.000000E+10  0.000000E+00  0.000000E+00  0.000000E+00  0.000000E+00  0.000000E+00  0.000000E+00  0.000000E+00  0.000000E+00  0.000000E+00  0.000000E+00  0.000000E+00  0.000000E+00       Funtag_D
      38  4.145803E+05  3.719507E+05       42629.7       14.5483     0.0648102      0.825420      0.189183      0.223770     0.0110551    -0.0100577    0.00840620       1.04527       Opttag_D
      38       85.7036       1.23626       1.00000  0.000000E+00       1.00000  0.000000E+00      0.939484       67.6175       1.00000       25.4531  0.000000E+00      -2.30947  0.000000E+00      -2.30947  0.000000E+00  0.000000E+00       4.61894       86.8527     0.0103683        1       Scatag_D
      38       1983.24       5958.60  0.000000E+00  0.000000E+00       2661.94  0.000000E+00       2616.36       7154.07  0.000000E+00       11415.8       46.1721       10793.5 -1.000000E+10 -1.000000E+10 -1.000000E+10 -1.000000E+10 -1.000000E+10  0.000000E+00  0.000000E+00  0.000000E+00  0.000000E+00  0.000000E+00  0.000000E+00  0.000000E+00  0.000000E+00  0.000000E+00  0.000000E+00  0.000000E+00  0.000000E+00       Funtag_D
      39  4.145472E+05  3.719132E+05       42634.0       9.06889     0.0657862      0.835471      0.189150      0.223808     0.0111412    -0.0103117    0.00840853       1.04518       Opttag_D
      39       85.7246       1.23647       1.00000  0.000000E+00       1.00000  0.000000E+00      0.939516       67.6402       1.00000       25.4450  0.000000E+00      -2.30989  0.000000E+00      -2.30989  0.000000E+00  0.000000E+00       4.61978       86.8527     0.0103683        1       Scatag_D
      39       1984.34       5957.53  0.000000E+00  0.000000E+00       2662.36  0.000000E+00       2617.29       7149.51  0.000000E+00       11420.5       46.2653       10796.2 -1.000000E+10 -1.000000E+10 -1.000000E+10 -1.000000E+10 -1.000000E+10  0.000000E+00  0.000000E+00  0.000000E+00  0.000000E+00  0.000000E+00  0.000000E+00  0.000000E+00  0.000000E+00  0.000000E+00  0.000000E+00  0.000000E+00  0.000000E+00       Funtag_D
      40  4.145178E+05  3.718710E+05       42646.8       7.39694     0.0668613      0.845750      0.189112      0.223846     0.0112381    -0.0106014    0.00841218       1.04524       Opttag_D
      40       85.7473       1.23662       1.00000  0.000000E+00       1.00000  0.000000E+00      0.939550       67.6631       1.00000       25.4357  0.000000E+00      -2.31023  0.000000E+00      -2.31023  0.000000E+00  0.000000E+00       4.62047       86.8527     0.0103683        1       Scatag_D
      40       1986.07       5958.21  0.000000E+00  0.000000E+00       2662.95  0.000000E+00       2617.84       7150.84  0.000000E+00       11425.8       46.4271       10798.7 -1.000000E+10 -1.000000E+10 -1.000000E+10 -1.000000E+10 -1.000000E+10  0.000000E+00  0.000000E+00  0.000000E+00  0.000000E+00  0.000000E+00  0.000000E+00  0.000000E+00  0.000000E+00  0.000000E+00  0.000000E+00  0.000000E+00  0.000000E+00       Funtag_D
      41  4.144932E+05  3.718297E+05       42663.5       9.10774     0.0680319      0.855840      0.189073      0.223893     0.0113328    -0.0109177    0.00841502       1.04530       Opttag_D
      41       85.7724       1.23693       1.00000  0.000000E+00       1.00000  0.000000E+00      0.939586       67.6862       1.00000       25.4265  0.000000E+00      -2.31141  0.000000E+00      -2.31141  0.000000E+00  0.000000E+00       4.62282       86.8527     0.0103683        1       Scatag_D
      41       1987.41       5958.94  0.000000E+00  0.000000E+00       2663.65  0.000000E+00       2618.31       7154.09  0.000000E+00       11432.4       46.6371       10802.1 -1.000000E+10 -1.000000E+10 -1.000000E+10 -1.000000E+10 -1.000000E+10  0.000000E+00  0.000000E+00  0.000000E+00  0.000000E+00  0.000000E+00  0.000000E+00  0.000000E+00  0.000000E+00  0.000000E+00  0.000000E+00  0.000000E+00  0.000000E+00       Funtag_D
      42  4.144642E+05  3.717761E+05       42688.1       9.44253     0.0697276      0.868824      0.189020      0.223968     0.0114560    -0.0113700    0.00841743       1.04566       Opttag_D
      42       85.8086       1.23751       1.00000  0.000000E+00       1.00000  0.000000E+00      0.939635       67.7166       1.00000       25.4144  0.000000E+00      -2.31357  0.000000E+00      -2.31357  0.000000E+00  0.000000E+00       4.62714       86.8527     0.0103683        1       Scatag_D
      42       1988.55       5962.99  0.000000E+00  0.000000E+00       2664.61  0.000000E+00       2618.93       7155.99  0.000000E+00       11442.2       46.9553       10807.9 -1.000000E+10 -1.000000E+10 -1.000000E+10 -1.000000E+10 -1.000000E+10  0.000000E+00  0.000000E+00  0.000000E+00  0.000000E+00  0.000000E+00  0.000000E+00  0.000000E+00  0.000000E+00  0.000000E+00  0.000000E+00  0.000000E+00  0.000000E+00       Funtag_D
      43  4.144386E+05  3.717462E+05       42692.5       9.16654     0.0706998      0.874242      0.188989      0.224015     0.0115246    -0.0116110    0.00841622       1.04575       Opttag_D
      43       85.8298       1.23788       1.00000  0.000000E+00       1.00000  0.000000E+00      0.939660       67.7297       1.00000       25.4075  0.000000E+00      -2.31609  0.000000E+00      -2.31609  0.000000E+00  0.000000E+00       4.63218       86.8527     0.0103683        1       Scatag_D
      43       1987.98       5964.00  0.000000E+00  0.000000E+00       2665.34  0.000000E+00       2618.73       7151.14  0.000000E+00       11447.0       47.0597       10811.2 -1.000000E+10 -1.000000E+10 -1.000000E+10 -1.000000E+10 -1.000000E+10  0.000000E+00  0.000000E+00  0.000000E+00  0.000000E+00  0.000000E+00  0.000000E+00  0.000000E+00  0.000000E+00  0.000000E+00  0.000000E+00  0.000000E+00  0.000000E+00       Funtag_D
      44  4.144131E+05  3.717232E+05       42689.9       8.08323     0.0713834      0.876494      0.188969      0.224047     0.0115774    -0.0117542    0.00841410       1.04574       Opttag_D
      44       85.8449       1.23807       1.00000  0.000000E+00       1.00000  0.000000E+00      0.939677       67.7343       1.00000       25.4027  0.000000E+00      -2.31860  0.000000E+00      -2.31860  0.000000E+00  0.000000E+00       4.63720       86.8527     0.0103683        1       Scatag_D
      44       1986.98       5963.87  0.000000E+00  0.000000E+00       2665.77  0.000000E+00       2618.46       7144.63  0.000000E+00       11449.8       47.0385       10813.4 -1.000000E+10 -1.000000E+10 -1.000000E+10 -1.000000E+10 -1.000000E+10  0.000000E+00  0.000000E+00  0.000000E+00  0.000000E+00  0.000000E+00  0.000000E+00  0.000000E+00  0.000000E+00  0.000000E+00  0.000000E+00  0.000000E+00  0.000000E+00       Funtag_D
      45  4.143869E+05  3.716870E+05       42699.8       9.93625     0.0723847      0.878673      0.188938      0.224089     0.0116604    -0.0119465    0.00841457       1.04595       Opttag_D
      45       85.8669       1.23826       1.00000  0.000000E+00       1.00000  0.000000E+00      0.939700       67.7384       1.00000       25.3959  0.000000E+00      -2.32211  0.000000E+00      -2.32211  0.000000E+00  0.000000E+00       4.64422       86.8527     0.0103683        1       Scatag_D
      45       1987.20       5966.34  0.000000E+00  0.000000E+00       2666.25  0.000000E+00       2618.51       7144.13  0.000000E+00       11453.5       46.9605       10817.0 -1.000000E+10 -1.000000E+10 -1.000000E+10 -1.000000E+10 -1.000000E+10  0.000000E+00  0.000000E+00  0.000000E+00  0.000000E+00  0.000000E+00  0.000000E+00  0.000000E+00  0.000000E+00  0.000000E+00  0.000000E+00  0.000000E+00  0.000000E+00       Funtag_D
      46  4.143630E+05  3.716501E+05       42712.9       9.79423     0.0732875      0.879220      0.188910      0.224111     0.0117453    -0.0120846    0.00842046       1.04607       Opttag_D
      46       85.8866       1.23824       1.00000  0.000000E+00       1.00000  0.000000E+00      0.939720       67.7383       1.00000       25.3902  0.000000E+00      -2.32503  0.000000E+00      -2.32503  0.000000E+00  0.000000E+00       4.65006       86.8527     0.0103683        1       Scatag_D
      46       1989.98       5967.71  0.000000E+00  0.000000E+00       2666.78  0.000000E+00       2618.82       7145.77  0.000000E+00       11456.4       46.8767       10820.6 -1.000000E+10 -1.000000E+10 -1.000000E+10 -1.000000E+10 -1.000000E+10  0.000000E+00  0.000000E+00  0.000000E+00  0.000000E+00  0.000000E+00  0.000000E+00  0.000000E+00  0.000000E+00  0.000000E+00  0.000000E+00  0.000000E+00  0.000000E+00       Funtag_D
      47  4.143397E+05  3.716256E+05       42714.1       7.60179     0.0738037      0.879276      0.188896      0.224115     0.0118016    -0.0121640    0.00842455       1.04605       Opttag_D
      47       85.8980       1.23813       1.00000  0.000000E+00       1.00000  0.000000E+00      0.939730       67.7389       1.00000       25.3872  0.000000E+00      -2.32604  0.000000E+00      -2.32604  0.000000E+00  0.000000E+00       4.65209       86.8527     0.0103683        1       Scatag_D
      47       1991.91       5967.45  0.000000E+00  0.000000E+00       2666.93  0.000000E+00       2619.34       7141.63  0.000000E+00       11458.5       46.8425       10821.5 -1.000000E+10 -1.000000E+10 -1.000000E+10 -1.000000E+10 -1.000000E+10  0.000000E+00  0.000000E+00  0.000000E+00  0.000000E+00  0.000000E+00  0.000000E+00  0.000000E+00  0.000000E+00  0.000000E+00  0.000000E+00  0.000000E+00  0.000000E+00       Funtag_D
      48  4.143074E+05  3.715695E+05       42737.9       9.24786     0.0753012      0.880166      0.188856      0.224129     0.0119302    -0.0124082    0.00843286       1.04628       Opttag_D
      48       85.9310       1.23827       1.00000  0.000000E+00       1.00000  0.000000E+00      0.939757       67.7484       1.00000       25.3801  0.000000E+00      -2.32884  0.000000E+00      -2.32884  0.000000E+00  0.000000E+00       4.65768       86.8527     0.0103683        1       Scatag_D
      48       1995.85       5970.10  0.000000E+00  0.000000E+00       2667.35  0.000000E+00       2621.17       7144.61  0.000000E+00       11466.8       46.8758       10825.1 -1.000000E+10 -1.000000E+10 -1.000000E+10 -1.000000E+10 -1.000000E+10  0.000000E+00  0.000000E+00  0.000000E+00  0.000000E+00  0.000000E+00  0.000000E+00  0.000000E+00  0.000000E+00  0.000000E+00  0.000000E+00  0.000000E+00  0.000000E+00       Funtag_D
      49  4.142937E+05  3.715348E+05       42759.0       12.6091     0.0766001      0.880495      0.188825      0.224160     0.0120100    -0.0126433    0.00843682       1.04639       Opttag_D
      49       85.9600       1.23880       1.00000  0.000000E+00       1.00000  0.000000E+00      0.939776       67.7593       1.00000       25.3759  0.000000E+00      -2.33180  0.000000E+00      -2.33180  0.000000E+00  0.000000E+00       4.66361       86.8527     0.0103683        1       Scatag_D
      49       1997.72       5971.33  0.000000E+00  0.000000E+00       2667.84  0.000000E+00       2622.37       7148.44  0.000000E+00       11475.3       46.9202       10829.0 -1.000000E+10 -1.000000E+10 -1.000000E+10 -1.000000E+10 -1.000000E+10  0.000000E+00  0.000000E+00  0.000000E+00  0.000000E+00  0.000000E+00  0.000000E+00  0.000000E+00  0.000000E+00  0.000000E+00  0.000000E+00  0.000000E+00  0.000000E+00       Funtag_D
      50  4.142769E+05  3.715343E+05       42742.7       6.83513     0.0766859      0.880208      0.188823      0.224160     0.0120112    -0.0126495    0.00843221       1.04617       Opttag_D
      50       85.9624       1.23893       1.00000  0.000000E+00       1.00000  0.000000E+00      0.939775       67.7618       1.00000       25.3756  0.000000E+00      -2.33217  0.000000E+00      -2.33217  0.000000E+00  0.000000E+00       4.66434       86.8527     0.0103683        1       Scatag_D
      50       1995.54       5968.79  0.000000E+00  0.000000E+00       2667.76  0.000000E+00       2622.19       7136.65  0.000000E+00       11476.4       46.9483       10828.4 -1.000000E+10 -1.000000E+10 -1.000000E+10 -1.000000E+10 -1.000000E+10  0.000000E+00  0.000000E+00  0.000000E+00  0.000000E+00  0.000000E+00  0.000000E+00  0.000000E+00  0.000000E+00  0.000000E+00  0.000000E+00  0.000000E+00  0.000000E+00       Funtag_D
      51  4.142591E+05  3.715237E+05       42735.4       5.33490     0.0773123      0.879544      0.188809      0.224174     0.0120353    -0.0127406    0.00842646       1.04604       Opttag_D
      51       85.9771       1.23943       1.00000  0.000000E+00       1.00000  0.000000E+00      0.939779       67.7695       1.00000       25.3741  0.000000E+00      -2.33407  0.000000E+00      -2.33407  0.000000E+00  0.000000E+00       4.66815       86.8527     0.0103683        1       Scatag_D
      51       1992.82       5967.37  0.000000E+00  0.000000E+00       2667.79  0.000000E+00       2622.07       7127.66  0.000000E+00       11481.6       47.0209       10829.0 -1.000000E+10 -1.000000E+10 -1.000000E+10 -1.000000E+10 -1.000000E+10  0.000000E+00  0.000000E+00  0.000000E+00  0.000000E+00  0.000000E+00  0.000000E+00  0.000000E+00  0.000000E+00  0.000000E+00  0.000000E+00  0.000000E+00  0.000000E+00       Funtag_D
      52  4.142453E+05  3.715093E+05       42736.0       6.13369     0.0780672      0.878524      0.188795      0.224189     0.0120685    -0.0128460    0.00842455       1.04595       Opttag_D
      52       85.9942       1.23990       1.00000  0.000000E+00       1.00000  0.000000E+00      0.939784       67.7752       1.00000       25.3730  0.000000E+00      -2.33612  0.000000E+00      -2.33612  0.000000E+00  0.000000E+00       4.67223       86.8527     0.0103683        1       Scatag_D
      52       1991.91       5966.35  0.000000E+00  0.000000E+00       2667.85  0.000000E+00       2621.86       7123.08  0.000000E+00       11487.6       47.0840       10830.3 -1.000000E+10 -1.000000E+10 -1.000000E+10 -1.000000E+10 -1.000000E+10  0.000000E+00  0.000000E+00  0.000000E+00  0.000000E+00  0.000000E+00  0.000000E+00  0.000000E+00  0.000000E+00  0.000000E+00  0.000000E+00  0.000000E+00  0.000000E+00       Funtag_D
      53  4.142264E+05  3.714671E+05       42759.3       15.5656     0.0801991      0.874529      0.188753      0.224222     0.0121653    -0.0131153    0.00842500       1.04591       Opttag_D
      53       86.0407       1.24094       1.00000  0.000000E+00       1.00000  0.000000E+00      0.939794       67.7819       1.00000       25.3734  0.000000E+00      -2.34132  0.000000E+00      -2.34132  0.000000E+00  0.000000E+00       4.68263       86.8527     0.0103683        1       Scatag_D
      53       1992.12       5965.88  0.000000E+00  0.000000E+00       2668.05  0.000000E+00       2621.04       7126.01  0.000000E+00       11504.8       47.2943       10834.0 -1.000000E+10 -1.000000E+10 -1.000000E+10 -1.000000E+10 -1.000000E+10  0.000000E+00  0.000000E+00  0.000000E+00  0.000000E+00  0.000000E+00  0.000000E+00  0.000000E+00  0.000000E+00  0.000000E+00  0.000000E+00  0.000000E+00  0.000000E+00       Funtag_D
      54  4.141988E+05  3.714505E+05       42748.2       6.98626     0.0811886      0.872121      0.188736      0.224236     0.0122034    -0.0132347    0.00842469       1.04540       Opttag_D
      54       86.0619       1.24144       1.00000  0.000000E+00       1.00000  0.000000E+00      0.939796       67.7825       1.00000       25.3749  0.000000E+00      -2.34354  0.000000E+00      -2.34354  0.000000E+00  0.000000E+00       4.68707       86.8527     0.0103683        1       Scatag_D
      54       1991.98       5960.03  0.000000E+00  0.000000E+00       2668.09  0.000000E+00       2620.58       7111.46  0.000000E+00       11512.8       47.4032       10835.9 -1.000000E+10 -1.000000E+10 -1.000000E+10 -1.000000E+10 -1.000000E+10  0.000000E+00  0.000000E+00  0.000000E+00  0.000000E+00  0.000000E+00  0.000000E+00  0.000000E+00  0.000000E+00  0.000000E+00  0.000000E+00  0.000000E+00  0.000000E+00       Funtag_D
      55  4.141840E+05  3.714409E+05       42743.0       5.33378     0.0813719      0.870481      0.188732      0.224230     0.0122254    -0.0132351    0.00842399       1.04519       Opttag_D
      55       86.0658       1.24129       1.00000  0.000000E+00       1.00000  0.000000E+00      0.939793       67.7766       1.00000       25.3760  0.000000E+00      -2.34400  0.000000E+00      -2.34400  0.000000E+00  0.000000E+00       4.68800       86.8527     0.0103683        1       Scatag_D
      55       1991.65       5957.67  0.000000E+00  0.000000E+00       2668.05  0.000000E+00       2620.00       7109.17  0.000000E+00       11513.7       47.4302       10835.3 -1.000000E+10 -1.000000E+10 -1.000000E+10 -1.000000E+10 -1.000000E+10  0.000000E+00  0.000000E+00  0.000000E+00  0.000000E+00  0.000000E+00  0.000000E+00  0.000000E+00  0.000000E+00  0.000000E+00  0.000000E+00  0.000000E+00  0.000000E+00       Funtag_D
      56  4.141640E+05  3.714191E+05       42744.9       6.95172     0.0819461      0.866698      0.188719      0.224219     0.0122755    -0.0132579    0.00842348       1.04483       Opttag_D
      56       86.0781       1.24110       1.00000  0.000000E+00       1.00000  0.000000E+00      0.939785       67.7654       1.00000       25.3791  0.000000E+00      -2.34546  0.000000E+00      -2.34546  0.000000E+00  0.000000E+00       4.69092       86.8527     0.0103683        1       Scatag_D
      56       1991.41       5953.59  0.000000E+00  0.000000E+00       2667.97  0.000000E+00       2619.24       7112.36  0.000000E+00       11517.6       47.5039       10835.3 -1.000000E+10 -1.000000E+10 -1.000000E+10 -1.000000E+10 -1.000000E+10  0.000000E+00  0.000000E+00  0.000000E+00  0.000000E+00  0.000000E+00  0.000000E+00  0.000000E+00  0.000000E+00  0.000000E+00  0.000000E+00  0.000000E+00  0.000000E+00       Funtag_D
      57  4.141503E+05  3.713948E+05       42755.5       12.8507     0.0827896      0.861411      0.188705      0.224207     0.0123312    -0.0132959    0.00842296       1.04454       Opttag_D
      57       86.0969       1.24110       1.00000  0.000000E+00       1.00000  0.000000E+00      0.939773       67.7530       1.00000       25.3834  0.000000E+00      -2.34767  0.000000E+00      -2.34767  0.000000E+00  0.000000E+00       4.69535       86.8527     0.0103683        1       Scatag_D
      57       1991.16       5950.25  0.000000E+00  0.000000E+00       2667.82  0.000000E+00       2618.90       7119.74  0.000000E+00       11524.0       47.5820       10836.1 -1.000000E+10 -1.000000E+10 -1.000000E+10 -1.000000E+10 -1.000000E+10  0.000000E+00  0.000000E+00  0.000000E+00  0.000000E+00  0.000000E+00  0.000000E+00  0.000000E+00  0.000000E+00  0.000000E+00  0.000000E+00  0.000000E+00  0.000000E+00       Funtag_D
      58  4.141301E+05  3.713840E+05       42746.1       7.48302     0.0831678      0.859144      0.188697      0.224204     0.0123562    -0.0133122    0.00842103       1.04429       Opttag_D
      58       86.1055       1.24112       1.00000  0.000000E+00       1.00000  0.000000E+00      0.939767       67.7484       1.00000       25.3855  0.000000E+00      -2.34871  0.000000E+00      -2.34871  0.000000E+00  0.000000E+00       4.69742       86.8527     0.0103683        1       Scatag_D
      58       1990.25       5947.42  0.000000E+00  0.000000E+00       2667.75  0.000000E+00       2618.94       7110.79  0.000000E+00       11527.1       47.5856       10836.3 -1.000000E+10 -1.000000E+10 -1.000000E+10 -1.000000E+10 -1.000000E+10  0.000000E+00  0.000000E+00  0.000000E+00  0.000000E+00  0.000000E+00  0.000000E+00  0.000000E+00  0.000000E+00  0.000000E+00  0.000000E+00  0.000000E+00  0.000000E+00       Funtag_D
      59  4.141091E+05  3.713691E+05       42740.0       6.26676     0.0838154      0.855594      0.188685      0.224203     0.0123904    -0.0133414    0.00841907       1.04406       Opttag_D
      59       86.1207       1.24130       1.00000  0.000000E+00       1.00000  0.000000E+00      0.939758       67.7430       1.00000       25.3884  0.000000E+00      -2.35040  0.000000E+00      -2.35040  0.000000E+00  0.000000E+00       4.70081       86.8527     0.0103683        1       Scatag_D
      59       1989.33       5944.73  0.000000E+00  0.000000E+00       2667.61  0.000000E+00       2619.12       7102.43  0.000000E+00       11532.6       47.5723       10836.6 -1.000000E+10 -1.000000E+10 -1.000000E+10 -1.000000E+10 -1.000000E+10  0.000000E+00  0.000000E+00  0.000000E+00  0.000000E+00  0.000000E+00  0.000000E+00  0.000000E+00  0.000000E+00  0.000000E+00  0.000000E+00  0.000000E+00  0.000000E+00       Funtag_D
      60  4.140913E+05  3.713533E+05       42738.0       6.85787     0.0845383      0.851608      0.188674      0.224198     0.0124266    -0.0133674    0.00841975       1.04386       Opttag_D
      60       86.1376       1.24149       1.00000  0.000000E+00       1.00000  0.000000E+00      0.939749       67.7361       1.00000       25.3916  0.000000E+00      -2.35201  0.000000E+00      -2.35201  0.000000E+00  0.000000E+00       4.70401       86.8527     0.0103683        1       Scatag_D
      60       1989.64       5942.54  0.000000E+00  0.000000E+00       2667.40  0.000000E+00       2619.34       7095.20  0.000000E+00       11538.9       47.5747       10837.4 -1.000000E+10 -1.000000E+10 -1.000000E+10 -1.000000E+10 -1.000000E+10  0.000000E+00  0.000000E+00  0.000000E+00  0.000000E+00  0.000000E+00  0.000000E+00  0.000000E+00  0.000000E+00  0.000000E+00  0.000000E+00  0.000000E+00  0.000000E+00       Funtag_D
      61  4.140853E+05  3.713182E+05       42767.0       19.0350     0.0862582      0.842654      0.188649      0.224186     0.0125071    -0.0134161    0.00842804       1.04380       Opttag_D
      61       86.1767       1.24188       1.00000  0.000000E+00       1.00000  0.000000E+00      0.939734       67.7156       1.00000       25.3998  0.000000E+00      -2.35490  0.000000E+00      -2.35490  0.000000E+00  0.000000E+00       4.70980       86.8527     0.0103683        1       Scatag_D
      61       1993.57       5941.81  0.000000E+00  0.000000E+00       2666.80  0.000000E+00       2620.33       7101.21  0.000000E+00       11554.8       47.7381       10840.8 -1.000000E+10 -1.000000E+10 -1.000000E+10 -1.000000E+10 -1.000000E+10  0.000000E+00  0.000000E+00  0.000000E+00  0.000000E+00  0.000000E+00  0.000000E+00  0.000000E+00  0.000000E+00  0.000000E+00  0.000000E+00  0.000000E+00  0.000000E+00       Funtag_D
      62  4.140541E+05  3.713144E+05       42739.7       5.95346     0.0864358      0.841718      0.188647      0.224185     0.0125159    -0.0134239    0.00842740       1.04347       Opttag_D
      62       86.1807       1.24191       1.00000  0.000000E+00       1.00000  0.000000E+00      0.939733       67.7128       1.00000       25.4004  0.000000E+00      -2.35516  0.000000E+00      -2.35516  0.000000E+00  0.000000E+00       4.71032       86.8527     0.0103683        1       Scatag_D
      62       1993.26       5938.09  0.000000E+00  0.000000E+00       2666.70  0.000000E+00       2620.43       7076.18  0.000000E+00       11556.1       47.7653       10841.2 -1.000000E+10 -1.000000E+10 -1.000000E+10 -1.000000E+10 -1.000000E+10  0.000000E+00  0.000000E+00  0.000000E+00  0.000000E+00  0.000000E+00  0.000000E+00  0.000000E+00  0.000000E+00  0.000000E+00  0.000000E+00  0.000000E+00  0.000000E+00       Funtag_D
      63  4.140455E+05  3.713097E+05       42735.7       4.26027     0.0864687      0.841452      0.188644      0.224180     0.0125266    -0.0134191    0.00842766       1.04343       Opttag_D
      63       86.1816       1.24180       1.00000  0.000000E+00       1.00000  0.000000E+00      0.939735       67.7098       1.00000       25.3999  0.000000E+00      -2.35512  0.000000E+00      -2.35512  0.000000E+00  0.000000E+00       4.71024       86.8527     0.0103683        1       Scatag_D
      63       1993.38       5937.65  0.000000E+00  0.000000E+00       2666.61  0.000000E+00       2620.34       7073.06  0.000000E+00       11555.7       47.8342       10841.1 -1.000000E+10 -1.000000E+10 -1.000000E+10 -1.000000E+10 -1.000000E+10  0.000000E+00  0.000000E+00  0.000000E+00  0.000000E+00  0.000000E+00  0.000000E+00  0.000000E+00  0.000000E+00  0.000000E+00  0.000000E+00  0.000000E+00  0.000000E+00       Funtag_D
      64  4.140327E+05  3.712951E+05       42737.6       5.57242     0.0868528      0.839534      0.188637      0.224169     0.0125602    -0.0134294    0.00843002       1.04339       Opttag_D
      64       86.1902       1.24166       1.00000  0.000000E+00       1.00000  0.000000E+00      0.939737       67.6999       1.00000       25.4007  0.000000E+00      -2.35563  0.000000E+00      -2.35563  0.000000E+00  0.000000E+00       4.71126       86.8527     0.0103683        1       Scatag_D
      64       1994.50       5937.11  0.000000E+00  0.000000E+00       2666.34  0.000000E+00       2620.69       7070.89  0.000000E+00       11557.8       48.0282       10842.3 -1.000000E+10 -1.000000E+10 -1.000000E+10 -1.000000E+10 -1.000000E+10  0.000000E+00  0.000000E+00  0.000000E+00  0.000000E+00  0.000000E+00  0.000000E+00  0.000000E+00  0.000000E+00  0.000000E+00  0.000000E+00  0.000000E+00  0.000000E+00       Funtag_D
      65  4.140143E+05  3.712732E+05       42741.1       8.33670     0.0874815      0.836810      0.188626      0.224156     0.0126105    -0.0134630    0.00843277       1.04332       Opttag_D
      65       86.2040       1.24153       1.00000  0.000000E+00       1.00000  0.000000E+00      0.939741       67.6843       1.00000       25.4024  0.000000E+00      -2.35687  0.000000E+00      -2.35687  0.000000E+00  0.000000E+00       4.71374       86.8527     0.0103683        1       Scatag_D
      65       1995.80       5936.34  0.000000E+00  0.000000E+00       2666.00  0.000000E+00       2621.82       7068.20  0.000000E+00       11560.2       48.2977       10844.5 -1.000000E+10 -1.000000E+10 -1.000000E+10 -1.000000E+10 -1.000000E+10  0.000000E+00  0.000000E+00  0.000000E+00  0.000000E+00  0.000000E+00  0.000000E+00  0.000000E+00  0.000000E+00  0.000000E+00  0.000000E+00  0.000000E+00  0.000000E+00       Funtag_D
      66  4.139947E+05  3.712384E+05       42756.3       9.42593     0.0888465      0.831610      0.188608      0.224143     0.0126903    -0.0135617    0.00844117       1.04327       Opttag_D
      66       86.2331       1.24155       1.00000  0.000000E+00       1.00000  0.000000E+00      0.939747       67.6552       1.00000       25.4084  0.000000E+00      -2.35920  0.000000E+00      -2.35920  0.000000E+00  0.000000E+00       4.71840       86.8527     0.0103683        1       Scatag_D
      66       1999.78       5935.80  0.000000E+00  0.000000E+00       2665.43  0.000000E+00       2624.89       7063.90  0.000000E+00       11567.0       48.7201       10850.8 -1.000000E+10 -1.000000E+10 -1.000000E+10 -1.000000E+10 -1.000000E+10  0.000000E+00  0.000000E+00  0.000000E+00  0.000000E+00  0.000000E+00  0.000000E+00  0.000000E+00  0.000000E+00  0.000000E+00  0.000000E+00  0.000000E+00  0.000000E+00       Funtag_D
      67  4.139770E+05  3.712292E+05       42747.8       5.79906     0.0892148      0.830667      0.188603      0.224140     0.0127115    -0.0135768    0.00844215       1.04320       Opttag_D
      67       86.2410       1.24158       1.00000  0.000000E+00       1.00000  0.000000E+00      0.939749       67.6474       1.00000       25.4104  0.000000E+00      -2.36007  0.000000E+00      -2.36007  0.000000E+00  0.000000E+00       4.72014       86.8527     0.0103683        1       Scatag_D
      67       2000.25       5935.03  0.000000E+00  0.000000E+00       2665.33  0.000000E+00       2625.58       7053.59  0.000000E+00       11567.8       48.6804       10851.6 -1.000000E+10 -1.000000E+10 -1.000000E+10 -1.000000E+10 -1.000000E+10  0.000000E+00  0.000000E+00  0.000000E+00  0.000000E+00  0.000000E+00  0.000000E+00  0.000000E+00  0.000000E+00  0.000000E+00  0.000000E+00  0.000000E+00  0.000000E+00       Funtag_D
      68  4.139604E+05  3.712192E+05       42741.3       5.73601     0.0896456      0.829907      0.188597      0.224138     0.0127345    -0.0135885    0.00844402       1.04313       Opttag_D
      68       86.2505       1.24167       1.00000  0.000000E+00       1.00000  0.000000E+00      0.939752       67.6385       1.00000       25.4127  0.000000E+00      -2.36117  0.000000E+00      -2.36117  0.000000E+00  0.000000E+00       4.72234       86.8527     0.0103683        1       Scatag_D
      68       2001.13       5934.25  0.000000E+00  0.000000E+00       2665.27  0.000000E+00       2625.97       7046.19  0.000000E+00       11568.0       48.5069       10852.0 -1.000000E+10 -1.000000E+10 -1.000000E+10 -1.000000E+10 -1.000000E+10  0.000000E+00  0.000000E+00  0.000000E+00  0.000000E+00  0.000000E+00  0.000000E+00  0.000000E+00  0.000000E+00  0.000000E+00  0.000000E+00  0.000000E+00  0.000000E+00       Funtag_D
      69  4.139482E+05  3.712071E+05       42741.1       6.88861     0.0902312      0.828943      0.188588      0.224134     0.0127623    -0.0135871    0.00844665       1.04313       Opttag_D
      69       86.2633       1.24178       1.00000  0.000000E+00       1.00000  0.000000E+00      0.939757       67.6272       1.00000       25.4161  0.000000E+00      -2.36242  0.000000E+00      -2.36242  0.000000E+00  0.000000E+00       4.72484       86.8527     0.0103683        1       Scatag_D
      69       2002.38       5934.16  0.000000E+00  0.000000E+00       2665.13  0.000000E+00       2626.04       7043.11  0.000000E+00       11569.2       48.3246       10852.8 -1.000000E+10 -1.000000E+10 -1.000000E+10 -1.000000E+10 -1.000000E+10  0.000000E+00  0.000000E+00  0.000000E+00  0.000000E+00  0.000000E+00  0.000000E+00  0.000000E+00  0.000000E+00  0.000000E+00  0.000000E+00  0.000000E+00  0.000000E+00       Funtag_D
      70  4.139327E+05  3.711934E+05       42739.3       6.14315     0.0909987      0.827994      0.188576      0.224126     0.0127936    -0.0135703    0.00844896       1.04305       Opttag_D
      70       86.2798       1.24196       1.00000  0.000000E+00       1.00000  0.000000E+00      0.939763       67.6127       1.00000       25.4207  0.000000E+00      -2.36369  0.000000E+00      -2.36369  0.000000E+00  0.000000E+00       4.72738       86.8527     0.0103683        1       Scatag_D
      70       2003.47       5933.26  0.000000E+00  0.000000E+00       2664.93  0.000000E+00       2625.41       7039.04  0.000000E+00       11571.6       48.1402       10853.5 -1.000000E+10 -1.000000E+10 -1.000000E+10 -1.000000E+10 -1.000000E+10  0.000000E+00  0.000000E+00  0.000000E+00  0.000000E+00  0.000000E+00  0.000000E+00  0.000000E+00  0.000000E+00  0.000000E+00  0.000000E+00  0.000000E+00  0.000000E+00       Funtag_D
      71  4.139181E+05  3.711843E+05       42733.8       5.48105     0.0914977      0.827812      0.188569      0.224114     0.0128146    -0.0135425    0.00844724       1.04294       Opttag_D
      71       86.2904       1.24205       1.00000  0.000000E+00       1.00000  0.000000E+00      0.939768       67.6033       1.00000       25.4236  0.000000E+00      -2.36438  0.000000E+00      -2.36438  0.000000E+00  0.000000E+00       4.72877       86.8527     0.0103683        1       Scatag_D
      71       2002.66       5932.00  0.000000E+00  0.000000E+00       2664.75  0.000000E+00       2624.10       7036.22  0.000000E+00       11573.3       48.0647       10852.7 -1.000000E+10 -1.000000E+10 -1.000000E+10 -1.000000E+10 -1.000000E+10  0.000000E+00  0.000000E+00  0.000000E+00  0.000000E+00  0.000000E+00  0.000000E+00  0.000000E+00  0.000000E+00  0.000000E+00  0.000000E+00  0.000000E+00  0.000000E+00       Funtag_D
      72  4.139010E+05  3.711688E+05       42732.2       8.68610     0.0925190      0.828021      0.188553      0.224086     0.0128502    -0.0134961    0.00844146       1.04287       Opttag_D
      72       86.3115       1.24233       1.00000  0.000000E+00       1.00000  0.000000E+00      0.939777       67.5860       1.00000       25.4296  0.000000E+00      -2.36559  0.000000E+00      -2.36559  0.000000E+00  0.000000E+00       4.73118       86.8527     0.0103683        1       Scatag_D
      72       1999.92       5931.28  0.000000E+00  0.000000E+00       2664.36  0.000000E+00       2621.76       7038.57  0.000000E+00       11576.9       48.0523       10851.4 -1.000000E+10 -1.000000E+10 -1.000000E+10 -1.000000E+10 -1.000000E+10  0.000000E+00  0.000000E+00  0.000000E+00  0.000000E+00  0.000000E+00  0.000000E+00  0.000000E+00  0.000000E+00  0.000000E+00  0.000000E+00  0.000000E+00  0.000000E+00       Funtag_D
      73  4.138869E+05  3.711636E+05       42723.3       6.43666     0.0929043      0.828661      0.188546      0.224079     0.0128621    -0.0135163    0.00843644       1.04270       Opttag_D
      73       86.3192       1.24249       1.00000  0.000000E+00       1.00000  0.000000E+00      0.939778       67.5806       1.00000       25.4315  0.000000E+00      -2.36618  0.000000E+00      -2.36618  0.000000E+00  0.000000E+00       4.73235       86.8527     0.0103683        1       Scatag_D
      73       1997.54       5929.29  0.000000E+00  0.000000E+00       2664.16  0.000000E+00       2621.28       7035.03  0.000000E+00       11577.8       48.1304       10850.1 -1.000000E+10 -1.000000E+10 -1.000000E+10 -1.000000E+10 -1.000000E+10  0.000000E+00  0.000000E+00  0.000000E+00  0.000000E+00  0.000000E+00  0.000000E+00  0.000000E+00  0.000000E+00  0.000000E+00  0.000000E+00  0.000000E+00  0.000000E+00       Funtag_D
      74  4.138776E+05  3.711619E+05       42715.7       4.84440     0.0929832      0.829182      0.188545      0.224079     0.0128661    -0.0135408    0.00843376       1.04257       Opttag_D
      74       86.3207       1.24256       1.00000  0.000000E+00       1.00000  0.000000E+00      0.939777       67.5805       1.00000       25.4314  0.000000E+00      -2.36644  0.000000E+00      -2.36644  0.000000E+00  0.000000E+00       4.73287       86.8527     0.0103683        1       Scatag_D
      74       1996.27       5927.82  0.000000E+00  0.000000E+00       2664.10  0.000000E+00       2621.63       7031.07  0.000000E+00       11577.3       48.1861       10849.3 -1.000000E+10 -1.000000E+10 -1.000000E+10 -1.000000E+10 -1.000000E+10  0.000000E+00  0.000000E+00  0.000000E+00  0.000000E+00  0.000000E+00  0.000000E+00  0.000000E+00  0.000000E+00  0.000000E+00  0.000000E+00  0.000000E+00  0.000000E+00       Funtag_D
      75  4.138642E+05  3.711551E+05       42709.1       5.37061     0.0934287      0.830627      0.188539      0.224079     0.0128816    -0.0136071    0.00843150       1.04241       Opttag_D
      75       86.3294       1.24286       1.00000  0.000000E+00       1.00000  0.000000E+00      0.939774       67.5774       1.00000       25.4327  0.000000E+00      -2.36753  0.000000E+00      -2.36753  0.000000E+00  0.000000E+00       4.73506       86.8527     0.0103683        1       Scatag_D
      75       1995.20       5926.04  0.000000E+00  0.000000E+00       2663.88  0.000000E+00       2623.19       7027.46  0.000000E+00       11576.4       48.2594       10848.7 -1.000000E+10 -1.000000E+10 -1.000000E+10 -1.000000E+10 -1.000000E+10  0.000000E+00  0.000000E+00  0.000000E+00  0.000000E+00  0.000000E+00  0.000000E+00  0.000000E+00  0.000000E+00  0.000000E+00  0.000000E+00  0.000000E+00  0.000000E+00       Funtag_D
      76  4.138597E+05  3.711473E+05       42712.4       9.85461     0.0941572      0.832149      0.188531      0.224078     0.0128996    -0.0136549    0.00843337       1.04250       Opttag_D
      76       86.3438       1.24327       1.00000  0.000000E+00       1.00000  0.000000E+00      0.939771       67.5706       1.00000       25.4365  0.000000E+00      -2.36877  0.000000E+00      -2.36877  0.000000E+00  0.000000E+00       4.73754       86.8527     0.0103683        1       Scatag_D
      76       1996.09       5927.01  0.000000E+00  0.000000E+00       2663.63  0.000000E+00       2624.54       7027.02  0.000000E+00       11576.7       48.1678       10849.2 -1.000000E+10 -1.000000E+10 -1.000000E+10 -1.000000E+10 -1.000000E+10  0.000000E+00  0.000000E+00  0.000000E+00  0.000000E+00  0.000000E+00  0.000000E+00  0.000000E+00  0.000000E+00  0.000000E+00  0.000000E+00  0.000000E+00  0.000000E+00       Funtag_D
      77  4.138504E+05  3.711455E+05       42704.9       4.77881     0.0941495      0.832183      0.188530      0.224076     0.0129035    -0.0136397    0.00843403       1.04244       Opttag_D
      77       86.3437       1.24320       1.00000  0.000000E+00       1.00000  0.000000E+00      0.939771       67.5702       1.00000       25.4365  0.000000E+00      -2.36886  0.000000E+00      -2.36886  0.000000E+00  0.000000E+00       4.73771       86.8527     0.0103683        1       Scatag_D
      77       1996.40       5926.29  0.000000E+00  0.000000E+00       2663.64  0.000000E+00       2624.60       7020.33  0.000000E+00       11576.3       48.1255       10849.2 -1.000000E+10 -1.000000E+10 -1.000000E+10 -1.000000E+10 -1.000000E+10  0.000000E+00  0.000000E+00  0.000000E+00  0.000000E+00  0.000000E+00  0.000000E+00  0.000000E+00  0.000000E+00  0.000000E+00  0.000000E+00  0.000000E+00  0.000000E+00       Funtag_D
      78  4.138428E+05  3.711395E+05       42703.3       3.37923     0.0944664      0.832860      0.188526      0.224072     0.0129173    -0.0136284    0.00843638       1.04250       Opttag_D
      78       86.3501       1.24324       1.00000  0.000000E+00       1.00000  0.000000E+00      0.939769       67.5660       1.00000       25.4384  0.000000E+00      -2.36948  0.000000E+00      -2.36948  0.000000E+00  0.000000E+00       4.73895       86.8527     0.0103683        1       Scatag_D
      78       1997.51       5926.99  0.000000E+00  0.000000E+00       2663.55  0.000000E+00       2625.10       7016.55  0.000000E+00       11575.9       48.0290       10849.6 -1.000000E+10 -1.000000E+10 -1.000000E+10 -1.000000E+10 -1.000000E+10  0.000000E+00  0.000000E+00  0.000000E+00  0.000000E+00  0.000000E+00  0.000000E+00  0.000000E+00  0.000000E+00  0.000000E+00  0.000000E+00  0.000000E+00  0.000000E+00       Funtag_D

   MaxLik Scaling Cycle 0083

   Wilson plot

   Statistics in resolution bins after refinement cycle 0003
```


|  |  |
| --- | --- |
|  | - Rxpct factors plot- Rxpct factors table- <Log-Lik Gain>- Ln Sigma A- Correlation Coefficients |


```
   Iters     Tot_Funct    Xray_Funct    Geom_Funct          Grms     rmsD2init     maxD2init             R         Rfree           LLG       LLGfree     rms(bond)    rms(angle)
   Iters             K   B_IMPF_FRAG        K_MISS   B_TEMP_MISS   K_IMPF_MISS   B_IMPF_MISS        K_SOLV   B_TEMP_SOLV   K_IMPF_SOLV   B_IMPF_SOLV        B_TEMP     B_TEMP_11     B_TEMP_12     B_TEMP_22     B_TEMP_13     B_TEMP_23     B_TEMP_33      K_WILSON      B_WILSON      FGCYCLES
   Iters          BOND         ANGLE      IMPROPER       TORSION    SINTORSION      TRIGONAL        PLANAR  BCORRELATION       CONTACT     IDEALDIST        CHIRAL    SIMILARITY       OCCSSUM  UTILDISTANCE     UTILANGLE   UTILTORSION        RHOFIT        ADP-01        ADP-02        ADP-03        ADP-04        ADP-05        ADP-06        ADP-07        ADP-08        ADP-09        ADP-10      RHOFITPC            QM
      79  4.138366E+05  3.711317E+05       42704.9       4.00113     0.0949235      0.833967      0.188519      0.224067     0.0129353    -0.0136242    0.00843879       1.04263       Opttag_D
      79       86.3591       1.24333       1.00000  0.000000E+00       1.00000  0.000000E+00      0.939767       67.5602       1.00000       25.4413  0.000000E+00      -2.37031  0.000000E+00      -2.37031  0.000000E+00  0.000000E+00       4.74062       86.8527     0.0103683        1       Scatag_D
      79       1998.65       5928.45  0.000000E+00  0.000000E+00       2663.44  0.000000E+00       2625.79       7014.62  0.000000E+00       11575.7       47.9703       10850.3 -1.000000E+10 -1.000000E+10 -1.000000E+10 -1.000000E+10 -1.000000E+10  0.000000E+00  0.000000E+00  0.000000E+00  0.000000E+00  0.000000E+00  0.000000E+00  0.000000E+00  0.000000E+00  0.000000E+00  0.000000E+00  0.000000E+00  0.000000E+00       Funtag_D
   Geometry Restraint Deviations SCREEN Information

 

 The refinement has converged on gradient RMS limit (-glim).

 No -bcorrel_zeroocchydrogens_factor flag found - keeping default: will set B for OCC=0 hydrogens to    1.200 times heavy-atom B


 TLS information. Total number of groups:     2


 Group   Natoms    FixRB   FixTLS   Group Tag    Group Spec
     1     2771      Y        Y     tls1    { A|* }
     2     2769      Y        Y     tls2    { B|* }
 
NOTE BUSTER_TLS_SET tls1 { A|* }
NOTE BUSTER_TLS_T   tls1     -3.26149E-01 -2.32596E-01  4.45630E-01 -3.91806E-02  3.95837E-02  4.09452E-02
NOTE BUSTER_TLS_L   tls1      1.68403E+00  1.68502E+00  1.03262E+00  3.16405E-01 -6.37610E-01  4.28922E-01
NOTE BUSTER_TLS_S   tls1      2.83610E-01 -2.29329E-01  2.89686E-01 -1.27619E-01 -5.00525E-02 -1.81171E-01  2.02995E-02 -1.16203E-01
NOTE BUSTER_TLS_O   tls1     -2.33284E+01 -8.36882E+01  1.12128E+01
NOTE BUSTER_TLS_FIX tls1 ALL
 
NOTE BUSTER_TLS_SET tls2 { B|* }
NOTE BUSTER_TLS_T   tls2     -3.93889E-01 -3.24897E-01  5.24628E-01  1.56106E-02  2.30904E-02 -3.24036E-02
NOTE BUSTER_TLS_L   tls2      1.80298E+00  1.67200E+00  9.38839E-01  7.03942E-01 -9.42582E-01 -8.62192E-01
NOTE BUSTER_TLS_S   tls2     -3.02785E-02  1.54983E-01 -1.28845E-01  1.24089E-01  2.68450E-01  6.71414E-02 -1.03280E-01  6.94089E-02
NOTE BUSTER_TLS_O   tls2      2.20360E+01 -7.19286E+01  3.53859E+00
NOTE BUSTER_TLS_FIX tls2 ALL

 For final position, Geometry function summary
     term                           number     weight       rms        GooF      function     fn/numb
     ================================================================================================
     BOND bond lengths (angs)       5655       2.000      0.0084       0.420      1998.65       0.353
     ANGL bond angles (degs)        7677       2.000        1.04       0.621      5928.45       0.772
     TORS split-harmonic tors       1267       0.000       18.84       1.186         0.00       0.000
     SINTOR sinusoidal tors         1267       2.000       18.84                  2663.44       2.102
     PLAN general plane (angs)       962       5.000      0.0148       0.739      2625.79       2.730
     BCORrelations (angs^2)         5644      10.000       0.988       0.353      7014.62       1.243
     CONT (bad contacts)               0       5.000                                 0.00
     IDEAL(ideal-dist contact)      5029       4.000                             11575.70
     CHIRAL (gelly semiharmon)       743       5.000                                47.97
     SIM similarity restraint for NCS and/or TARGET                              10850.29
     OCCSUM restraint on sum of occupanies                                           0.00       as none defined
     DISTAN utility restraints interatomic distance                                  0.00       as none defined
     UTILANGLE utility angle restraints                                              0.00       as none defined
     UTILTOR utility restraints on torsion angles                                    0.00       as none defined
     Peptide omega torsion angles                           2.96 degs - monitor only

 Weighted rms      0.0084         1.043  (temporary rms bond and angle for BUSTER to pick up!)
 Weighted intra-residual rms(bond) for StdProtein (excluding H)      0.0082
 Weighted intra-residual rms(bond) for Buffer     (excluding H)      0.0030
 Outputing geometry progress graph in plotmtv format to file:Geom_Res.mtv

 ############### Running final geometry/screen analysis (screen_final.txt)

 s/r gelly normal termination
```

Statistics and scale factors vs. cycle number
```

|  |  |  |
| --- | --- | --- |
|  | - Rxpct factors- <Log-Lik Gain>- GEOM Residuals- Ln Sigma_A_ | - Scale Factors- B Factors- Imperfection Factors |

```
 Writing F_calc(model+solvent) to complete.mtz

   Final Geometry Information


---


### 2. Final results

Statistics and scale factors vs. cycle number
```

|  |  |  |
| --- | --- | --- |
|  | - Rxpct factors- <Log-Lik Gain>- GEOM Residuals- Ln Sigma_A_ | - Scale Factors- B Factors- Imperfection Factors |

```
   Average Figure of Merit
   Structure Factor Amplitudes


 # normal termination

  Program stopped.
```

closesok message: time spent in tree = 1031.57 (user) 36.32 (sys)

---

# Normal termination

```
 Resources usage summary:
     Time spent in client ...................  1031.58 (user)       36.34 (system)
     Time spent in scripts (    27 calls) ...     1.85 (user)        0.12 (system)
     Time spent in server ...................     0.00 (user)        0.01 (system)

     Max resident memory used (kb) .......... 437412
     Number of page faults .................. 0 (major)    268484 (minor)
     Number of context switches ............. 1107 (niced)    4128 (other)
```
